# Poly(thioctic acid): From Bottom‐Up Self‐Assembly to 3D‐Fused Deposition Modeling Printing

**DOI:** 10.1002/advs.202203630

**Published:** 2022-10-11

**Authors:** Changyong Cai, Shuanggen Wu, Yunfei Zhang, Fenfang Li, Zhijian Tan, Shengyi Dong

**Affiliations:** ^1^ Department of Organic Chemistry College of Chemistry and Chemical Engineering Hunan University Changsha 410082 China; ^2^ Department of Pharmaceutical Engineering College of Chemistry and Chemical Engineering Central South University Changsha 410083 China; ^3^ Institute of Bast Fiber Crops Chinese Academy of Agricultural Sciences Changsha 410205 China

**Keywords:** 3D‐fused deposition modeling printing, bottom‐up self‐assembly, dynamical polymerization, supramolecular additive manufacturing, thioctic acid

## Abstract

Inspired by the bottom‐up assembly in nature, an artificial self‐assembly pattern is introduced into 3D‐fused deposition modeling (FDM) printing to achieve additive manufacturing on the macroscopic scale. Thermally activated polymerization of thioctic acid (**TA**) enabled the bulk construction of poly(**TA**), and yielded unique time‐dependent self‐assembly. Freshly prepared poly(**TA**) can spontaneously and continuously transfer into higher‐molecular‐weight species and low‐molecular‐weight **TA** monomers. Poly(**TA**) and the newly formed **TA** further assembled into self‐reinforcing materials via microscopic‐phase separation. Bottom‐up self‐assembly patterns on different scales are fully realized by 3D FDM printing of poly(**TA**): thermally induced polymerization of **TA** (microscopic‐scale assembly) to poly(**TA**) and 3D printing (macroscopic‐scale assembly) of poly(**TA**) are simultaneously achieved in the 3D‐printing process; after 3D printing, the poly(**TA**) modes show mechanically enhanced features over time, arising from the microscopic self‐assembly of poly(**TA**) and **TA**. This study clearly demonstrates that micro‐ and macroscopic bottom‐up self‐assembly can be applied in 3D additive manufacturing.

## Graphical abstract

Naturally inspired bottom‐up self‐assembly patterns on different scales are fully realized by 3D‐fused deposition modeling printing of poly(thioctic acid): thermally induced polymerization of thioctic acid to poly(thioctic acid) and 3D printing of poly(thioctic acid) are simultaneously achieved in the 3D‐printing process. After 3D printing, the poly(thioctic acid) modes show mechanically enhanced features over time, arising from the microscopic self‐assembly of poly(thioctic acid) and thioctic acid.

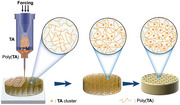

## 1 Introduction

Bottom‐up assembly is a universal phenomenon in nature that occurs from the microscale (≈nm) to the macroscale (≈cm).^[^ ^1^ ^]^ In biological systems, assembly activities on different scales are closely related and occur simultaneously.^[^ ^2^ ^]^ After the formation of functional aggregates on the large‐scale, microscopic self‐assembly has been shown to profoundly affect the structure and function of materials.^[^ ^3^ ^]^ Inspired by nature, chemistry and materials scientists have exploited bottom‐up assembly patterns in synthesizing new structures and materials.^[^ ^4^ ^]^ Chemical self‐assembly of small molecules and 3D printing of polymers are two typical examples of artificial bottom‐up assemblies on the microscopic and macroscopic scales, respectively.^[^ ^5^ ^]^

Recently, 3D printing of supramolecular materials, with hydrogels as the main object, has been realized.^[^ ^6^ ^]^ 3D printing, as a new processing technology, has been applied in fabricating supramolecular materials with designed shapes and sizes.^[^ ^7^ ^]^ However, chemical self‐assembly was achieved prior to 3D printing. In other words, chemical assembly and 3D printing are two independent techniques for the construction of bulk materials, and differ from the assembly pattern in nature.

To mimic the bottom‐up assembly in nature by chemical self‐assembly (microscopic scale) and 3D printing (macroscopic scale), self‐assembly should be the driving force for 3D printing. Therefore, the assembly behavior should be thermally initialized (for fused deposition modeling, FDM) or efficiently induced by UV irradiation (for stereolithography appearance, SLA).^[^ ^8^ ^]^ Moreover, time‐dependent bottom‐up assembly is preferred, especially after the 3D‐printing process.

Herein, we report the bottom‐up chemical assembly and 3D printing of a natural low‐molecular‐weight monomer, thioctic acid (**TA**). Thermally activated dynamical polymerization of **TA** yields poly(**TA**), which exhibits a unique time‐dependent self‐reinforcing pattern. Thus, **TA** can be used as the starting material in the FDM 3D printing. After 3D printing, the mechanical capacity and rigidity of the poly(**TA**) modes increased continuously via dynamic polymerization‐induced microscopic self‐assembly of poly(**TA**) and **TA**.

## 2 Results and Discussion

### 2.1 Time‐Dependent Assembly Pattern of Poly(**TA**)

**Figure 1:**
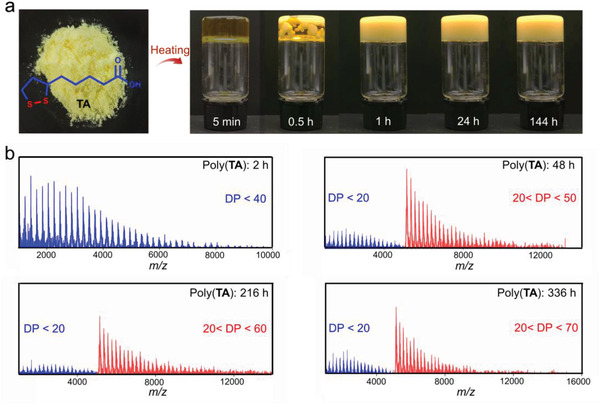
Time‐dependent assembly of poly(**TA**). a) Time‐dependent process photos of poly(**TA**). b) Time‐dependent MALDI‐TOF‐MS spectra of poly(**TA**).

Owing to the disulfide bond in **TA**, yellow **TA** powders can easily and rapidly undergo ring‐open polymerization upon heating to yield poly(**TA**), which is a translucent material that becomes opaque after standing at room temperature (**Figure** **1**a).^[^ ^9^ ^]^ Compared with the partially crystalline nature of **TA**, freshly prepared poly(**TA**) has an amorphous and highly compact structure (Table S1 and Figure S5, Supporting Information).

The time‐dependent self‐assembly pattern of poly(**TA**) was first revealed using matrix‐assisted laser desorption/ionization time‐of‐flight mass spectrometry (MALDI‐TOF‐MS) (Figure 1b and Figure S1, Supporting Information).^[^ ^9e^ ^]^ Polymeric structures with degrees of polymerization (DP) < 40 were found in freshly prepared poly(**TA**). After standing at 25 °C for 24 h, clear changes were observed in the mass spectra of poly(**TA**). Species with higher molecular weights and DP were found. For example, after storage under ambient conditions (25 °C, 50% RH) for 48 h, poly(**TA**) species with two distinct types of molecular‐weight distributions (DP < 20 and 20 < DP < 50) were detected in the mass spectrum of the poly(**TA**) sample. These results demonstrate that after the thermally induced polymerization of **TA**, poly(**TA**) undergoes the further assembly process.

Quantitative time‐dependent analysis was acquired for the poly(**TA**) samples. The majority of the **TA** monomers (>95%) were transformed into polymeric structures after heating, according to the high‐performance liquid chromatography (HPLC) measurements. An obvious time‐dependent reproduction of **TA** was observed after the fabrication of poly(**TA**). The content of **TA** reached a plateau at 54% after 15 days (Figures S2 and S3, Supporting Information). Infrared spectroscopy (IR) and PXRD measurements of the poly(**TA**) samples stored for different times clearly showed that peaks belonging to **TA** were found in the spectra of these samples, compared with the freshly prepared poly(**TA**), indicating the existence of a large amount of **TA** molecules in poly(**TA**) samples (Figures S4–S6, Supporting Information). According to the results from MALDI‐TOF‐MS and HPLC, it is clear that poly(**TA**) can be transferred to **TA** monomers and polymers with more repeated units over time after the initial polymerization. The model reactions in poly(**TA**) were simulated to reveal the mechanism of the time‐dependent self‐assembly of poly(**TA**). Heating was found to be favorable for the dimerization of **TA** (Δ*G* = 0.91 kcal mol^−1^). However, the dimers can spontaneously polymerize into higher‐molecular‐weight species (Δ*G* = −17.06 kcal mol^−1^), during which **TA** is simultaneously generated. The simulation results are consistent with the experimental observations (Figure S9 and Table S2, Supporting Information).

From the combined experimental and theoretical investigations, it is clear that the self‐assembly of **TA** involves two sequential stages: (a) thermally induced polymerization of **TA** to poly(**TA**) and (b) spontaneous depolymerization and repolymerization of poly(**TA**) to higher‐molecular‐weight polymers and low‐molecular‐weight **TA**. The stage (a) is thermally triggered. While the stage (b) is spontaneous and time‐dependent. Although various characterizations have confirmed the continuous supply of **TA** in poly(**TA**) materials, the influence of **TA** on the morphology of poly(**TA**) remains ambiguous. Thus, focus was then placed on the time‐dependent morphological behavior of poly(**TA**).

### 2.2 Time‐Dependent Morphological Changes in Poly(**TA**)

**Figure 2:**
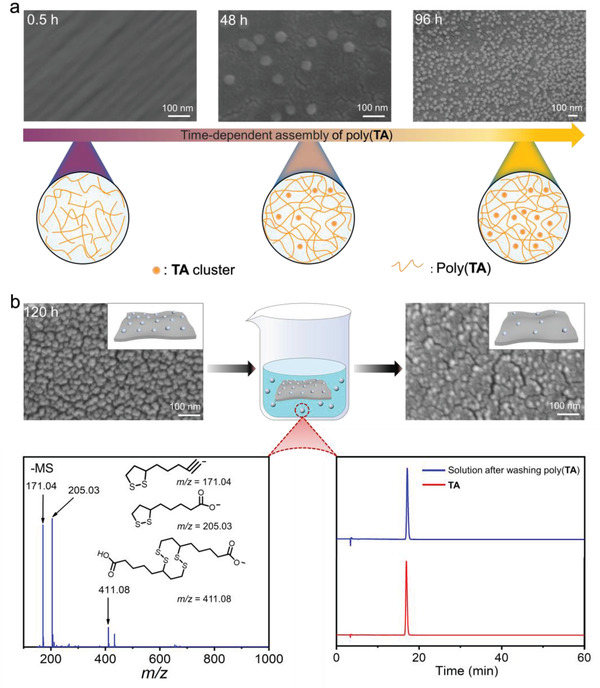
Time‐dependent morphological changes in poly(**TA**). a) Time‐dependent SEM images of poly(**TA**). b) Characterization of long‐time stored poly(**TA**).

This unique self‐assembly pattern was found to exert a significant influence on the morphology of poly(**TA**). For freshly prepared poly(**TA**), only compact and uniform structures were observed in the scanning electron microscope (SEM) images (**Figure** **2**a). A few cyclic structures with an average diameter of 40–60 nm were found in the poly(**TA**) sample that had been maintained at 25 °C for 48 h. The distribution of the cyclic structures exhibits a clear time dependence, as shown in Figure 2a and Figures S10–S15 (Supporting Information). After standing for 14 days, the entire tested samples of poly(**TA**) were full with cyclic structures. No changes in the shapes and sizes of the newly emerging structures were observed by comparing the SEM images of the poly(**TA**) samples obtained at different times. These observations raised two questions: What is the chemical composition of the cyclic structures? What is the influence of the structures?

The newly emerging structures are water‐soluble and could be flushed with water, as shown in Figure 2b. This information indicates that the cyclic structures were composed of **TA,** because **TA** is soluble, and poly(**TA**) is water insoluble. Electron spray ionization (ESI) mass, HPLC, and ^1^H NMR spectra analysis of the newly formed structures strongly supported this hypothesis (Figure 2c and Figure S16, Supporting Information). The small‐angle X‐ray scattering (SAXS) data indicated the absence of ordered structures in poly(**TA**), demonstrating that the aggregation of **TA** monomers does not occur in an ordered manner (Figure S17, Supporting Information). Simulation of the **TA** clusters clearly confirmed the irregular assembly of **TA** at the microscale, with hydrogen bonds and van der Waals interactions as the main driving forces (Table S3, Supporting Information).

Experimental and theoretical studies have demonstrated that the continuously emerging poly(**TA**) and **TA** exhibit a new self‐assembly pattern: **TA** monomers favor aggregation and realize (micro)phase separation with poly(**TA**) units. The presence of poly(**TA**) and **TA** is not due to a simple repolymerization process. Poly(**TA**) and **TA** are formed via dynamic self‐assembly of poly(**TA**). However, discovering the bottom‐up assembly of poly(**TA**) represents only half of the picture. Attention was shifted to studying the effect of this unique self‐assembly pattern on the mechanical properties of poly(**TA**).

### 2.3 Mechanical Properties of Poly(**TA**)

**Figure 3:**
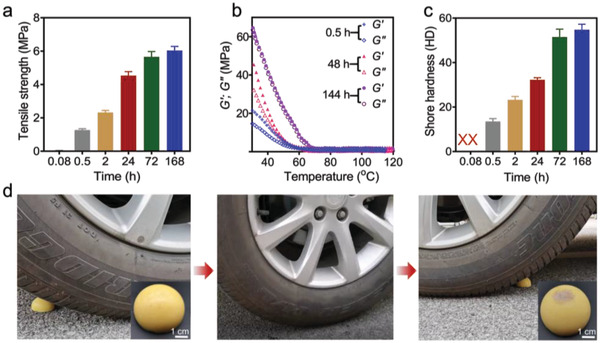
Mechanical properties of poly(**TA**). a) Time‐dependent tensile strength of poly(**TA**). b) Time‐dependent DMA of poly(**TA**). c) Time‐dependent Shore hardness (Shore D) of poly(**TA**). d) Photos of weight‐compression test of poly(**TA**) (a poly(**TA**) hemisphere: 38 mm in diameter, stored for 60 days; weight of the car: 1600 kg). Data in (a) and (c) are means ± S.D., *n* = 3.

Various time‐dependent measurements verified the importance of the self‐assembly of poly(**TA**) and **TA** in realizing the unique mechanical feature (**Figure** **3**a and Figures S18–S19, Supporting Information). Freshly prepared poly(**TA**) is a soft material with low tensile strength (0.029 MPa). After standing at 25 °C for 24 h, a tensile strength of 4.53 MPa was recorded, which is more than 150 times of that obtained from the fresh sample, indicating an obvious self‐enhancement of poly(**TA**). As time passed, the mechanical strength of poly(**TA**) increased, with the tensile strength reaching a maximum of 6.03 MPa.

The time‐dependent rheological behavior of poly(**TA**) was investigated. It is clear that the storage/loss moduli (*G*’ and *G*″) of freshly obtained poly(**TA**) are much lower than those of the long‐standing samples (Figure S20, Supporting Information). Dynamic thermomechanical analysis (DMA) tests provided more information: the *G*’ value of poly(**TA**) (after 24 h, 2.1 × 10^7^ Pa) was only 1/3 of that of poly(**TA**) at 10 days (6.3 × 10^7^ Pa), demonstrating that the mechanical strength of poly(**TA**) was enhanced over time (Figure 3b and Figure S21, Supporting Information). For the freshly prepared poly(**TA**), the Shore hardness (Shore D) was undetectable. After storage at 25 °C for 0.5 and 24 h, the Shore hardness reached 13.5 and 32.3 HD, respectively. Further extending the storage time significantly increased the hardness of poly(**TA**). For example, when poly(**TA**) was maintained at 25 °C for 7 days, the Shore hardness reached 54.7 HD, which is comparable to that of plastic materials (Shore hardness > 42) (Figure 3c).^[^ ^10^ ^]^ Meanwhile, after 7 days, the changes in the structures and mechanical properties of poly(**TA**) reached an equilibrium, which has been confirmed by TOF‐MS, mechanical strength tests, and SEM. Macroscopic tests further confirmed the excellent mechanical properties of poly(**TA**). As shown in Figure 3d and Video S1 (Supporting Information), after pressing by a car with the weight of 1600 kg, no reformations or fractures were found in the poly(**TA**) material (cast in a hemispheric shape, stored under ambient conditions for 30 days). When a poly(**TA**) ball was thrown on the floor (from 2.3 m height) more than 1000 times, the structural integrity and elasticity of the poly(**TA**) ball were perfectly maintained (Video S2, Supporting Information).

**Figure 4:**
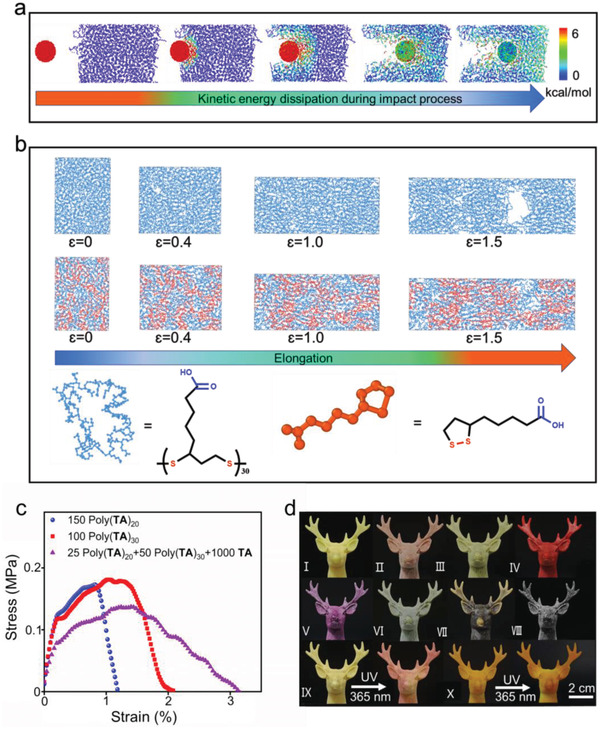
Molecular dynamics investigation of poly(**TA**). a) Energy dissipation process of poly(**TA**) under impact from MD simulations. b) Structure evolution of poly(**TA**) during the elongation process. c) Stress–strain of three poly**(TA)**/**TA** samples by large‐scale atomic/molecular massively parallel simulator (LAMMPS). d) Poly(**TA**) models with different additives [I: no additive; II: nickel chloride hexahydrate (5 wt%); III: azure B (5 wt%); IV: sudan II, BS (5 wt%); V: acid fuchsin sodium salt (5 wt%); VI: gallium (20 wt%); VII: cobalt chloride (5 wt%); VIII: magnetic Fe_3_O_4_ nanoparticles (20 wt%); IX: photochromic material (20 wt%); X: 1,4‐bis‐(*α*‐cyano‐4‐methoxystyryl)‐2,5‐dimethoxybenzene (0.1 wt%).

Simulation was applied to study the relationship between the self‐assembly pattern and self‐reinforcing behavior of poly(**TA**). The mechanism of energy dissipation in poly(**TA**) was intuitively revealed by simulating the shock resistance of poly(**TA**) (**Figure** **4**a; Figure S22, Table S3, and Video S3, Supporting Information). Collision among the poly(**TA**)/**TA** units enables rapid energy exchange with neighboring units to effectively disperse the force over a large area.^[^ ^11^ ^]^ Meanwhile, friction among the polymeric structures and elastic deformation of the bulk material also respond to fast energy dissipation.

More simulations were performed to study the relationship between the unique self‐assembly and mechanical strength of poly(**TA**) at the molecular and aggregated levels. Simulations with different numbers of poly(**TA**) molecules and **TA** were designed. As shown in Figure 4b,c, increasing the molecular weight of poly(**TA**) (without **TA**) only slightly enhanced the mechanical strength of poly(**TA**). However, incorporating **TA** monomers into poly(**TA**) with relatively few repeated units attenuated its mechanical strength. Simultaneously increasing the number of repeated units in poly(**TA**) and **TA** effectively strengthened the stress and strain capacities of poly(**TA**). The coexistence of **TA** significantly increased the nonbonding energy and stability of the poly(**TA**)/**TA** systems (Table S3, Supporting Information). Simulation of the tensile behavior of poly(**TA**) further confirmed the importance of **TA** in realizing excellent antistretching capacity (Figure 4b,c and Video S4, Supporting Information). These observations are consistent with the results of the mechanical tests.^[^ ^12^ ^]^

Owing to the thermo‐/time‐sensitive assembly behavior of poly(**TA**), excellent processability of poly(**TA**) was realized, which is the basis of 3D FDM‐printing technology. Poly(**TA**) was not only easily cast into different modes with high resolutions but also showed good compatibility with a variety of additives. As depicted in Figure 4d, organic/inorganic dyes, fluorescent materials, liquid metal, and nanoparticles were uniformly dispersed in the poly(**TA**) matrixes. Long‐term standing tests (12 months) confirmed the stability of the modified poly(**TA**) materials. The stimuli‐responsiveness of the additives in poly(**TA**) was fully realized by irradiation or by applying a magnetic field. In addition to poly(**TA**), **TA** powders also have good hot‐workability and can be directly processed into models with desired shapes and sizes by heating. Time‐dependent mechanical properties were fully observed in the poly(**TA**) or **TA** cast modes (Figure S23, Supporting Information), indicating that these additives do not interfere with the self‐assembly behavior of poly(**TA**)/**TA**.

### 2.4 3D Printing of Poly(**TA**) or **TA**

**Figure 5:**
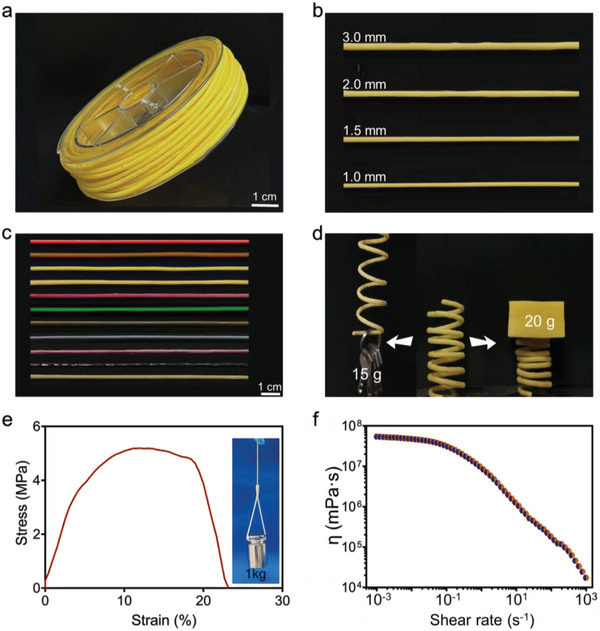
Filaments of poly(**TA**). a) Poly(**TA**) filament (1.5 mm). b) Poly(**TA**) filaments with different diameters. c) Poly(**TA**) filaments with different additives (1.5 mm) [from to bottom: sudan II, BS (5 wt%); 1,4‐bis‐(*α*‐cyano‐4‐methoxystyryl)‐2,5‐dimethoxybenzene (0.1 wt%); 1,4‐bis‐(*α*‐cyano‐4‐methoxystyryl)‐2,5‐dimethoxybenzene (0.1 wt%) by UV (365 nm); photochromic material (20 wt%); photochromic material (20 wt%) with UV irradiation (365 nm); long afterglow material SrAl_2_O_4_:Eu (10 wt%); cobalt chloride (5 wt%); azure B (5 wt%); acid fuchsin sodium salt (5 wt%); magnetic Fe_3_O_4_ nanoparticles (20 wt%); gallium (20 wt%)]. d) Poly(**TA**) spring in the weight loading/unloading test. e) Mechanical properties of poly(**TA**) filament. f) Shear rate‐dependent viscosity of poly(**TA**) (100 °C).

There are two main stages in 3D FDM‐printing: preparation of 3D printable materials and the 3D‐printing procedure. In the first stage, two types of materials are available, viscous melts and filaments. Hence, before 3D FDM printing, melts and filaments of poly(**TA**) were prepared (**Figure** **5**a; Figures S24–S25 and Video S5, Supporting Information). Poly(**TA**) exhibited typical temperature‐dependent viscosity, demonstrating that viscous melts can be obtained by simply heating poly(**TA**) (Figure S26, Supporting Information). Meanwhile, the **TA** powder can be directly transformed into viscous poly(**TA**) melts by heating for a short time (120 °C, 2–3 s). Compared with viscous melts, filaments are more common in 3D FDM printing. Long, flexible, and tough poly(**TA**) filaments with diameters of 1.0–3.0 mm were successfully obtained using a commercial injection molding machine (Figure 5b). Filaments with different additives were fabricated using the same method (Figure 5c). Poly(**TA**) filaments showed excellent flexibility. A spring was constructed from a long poly(**TA**) filament (22.5 cm in length, 2.0 mm in diameter). Repeated compression and tensile phenomena were observed during the weight loading/unloading tests (Figure 5d and Video S6, Supporting Information).

The poly(**TA**) filaments exhibited high mechanical strengths after standing at 25 °C (Figure 5e). For example, no fractures were observed in the long‐term (30 days) weight (1.0 kg) loading test of a thin poly(**TA**) filament (2.0 mm in diameter). A Young's modulus of 145.7 MPa was recorded for the poly(**TA**) filaments (stored for 7 days at 25 °C). Filaments directly obtained by heating **TA** powders showed similar mechanical performance. As shown in Figure 5f, the shear rate‐dependent viscosity of poly(**TA**) is measured at 100 °C, displaying the typical shear‐thinning behavior (from 5.4 × 10^7^ to 1.7 × 10^4^ mPa⋅s).^[^ ^13^ ^]^

**Figure 6:**
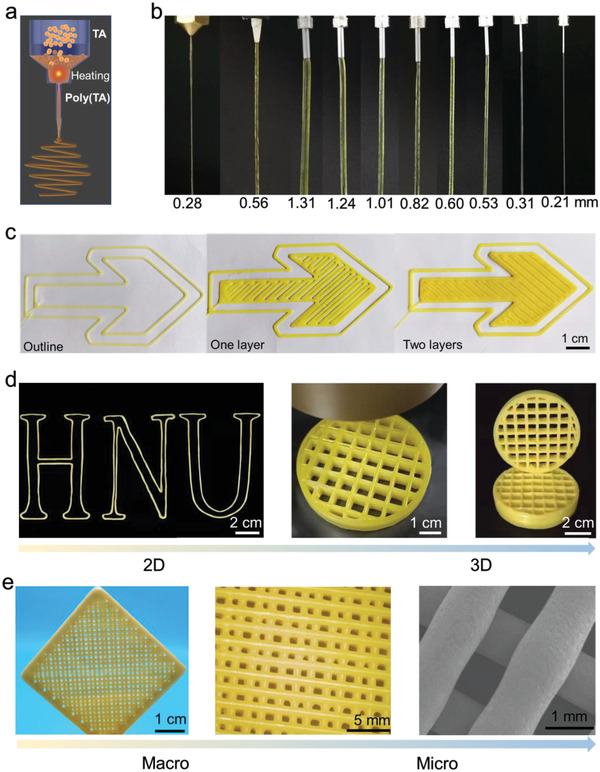
3D printing of poly(**TA**) and characterization of 3D‐printed models of poly(**TA**). a) Cartoon representation of 3D‐printing process. b) Photos of poly(**TA**) fibers. c) Layer‐by‐layer 3D printing (100 °C). **d)** Photos of 3D‐printed models. **e)** Macro–micro images of 3D‐printed models.

Poly(**TA**) or **TA** powders were employed in 3D printing using commercially available 3D printers, respectively. As shown in **Figure** **6**, Figure S27 and Video S7 (Supporting Information), thin, flexible, and translucent poly(**TA**) fibers were smoothly squeezed from the extruder nozzles (filament and viscous melt‐printing methods). The printing temperature plays an important role in the 3D printing of poly(**TA**) or **TA**.^[^ ^8^, ^14^ ^]^ A low temperature (60–80 °C) leads to failure in the extrusion process, while a high temperature (110–120 °C) increases the difficulty of molding (Figure S28, Supporting Information). The optimal temperature for the 3D printing of poly(**TA**) was 95–100 °C. To realize a fast‐curing process in the printed mode, a cooling device or cooling spray is preferred. Models were printed layer by layer in the 3D printing (Figure 6c), which was similar to the method of printing commercial materials (polylactic acid [PLA], polyamide [PA], acrylonitrile butadine styrene copolymers [ABS], and thermoplastic urethane [TPU]).^[^ ^15^ ^]^ It was possible to distinguish between different layers and printed edges in the printed modes. 2D or 3D models with sizes smaller than 0.5 × 0.5 × 0.5 cm^3^ or larger than 10.0 × 10.0 × 3.0 cm^3^ were rapidly printed on the substrate surfaces by the filament‐printing method or viscous melts method (Figure 6d; Figures S29–S32 and Videos S8–S9, Supporting Information). With the 3D printing of a hollow cylinder as an example, this poly(**TA**) mode has diameter of 6.08 cm and height of 1.37 cm, where 19.52 g of **TA** was used in the printing process. This mode was printed within 1.7 h at a working temperature of 100 °C (Figure 6d).

**Figure 7:**
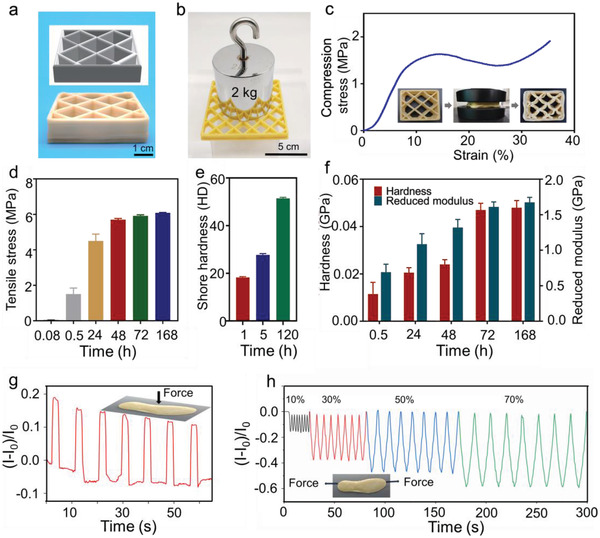
Mechanical parameters and applications of 3D‐printed models of poly(**TA**). a) 3D‐printed hollow box. b) Weight loading test of hollow box. c) Compression curve of the 3D‐printed hollow box. d) Time‐dependent tensile strength of poly(**TA**) fiber. e) Time‐dependent Shore hardness (Shore D) of 3D‐printed sample. f) Hardness and reduced modulus of the 3D‐printed sample by nanoindentation. g) Relative current variation of the pressure sensor in multiple cycles of loading/unloading tests (732.5 Pa). h) Relative current variation of the strain sensor in multiple cycles of loading/unloading tests. Data in c–d are means ± S.D., *n* = 3.

The printed poly(**TA**) modes were first subjected to morphological analysis: the resolution of 3D‐printed poly(**TA**) could reach as low as 0.2–1.3 mm, which is comparable to that of commercially available materials; no cobwebbing phenomenon and other fractures were observed during the entire printing process and after printing by optical microscope and SEM (Figure 6e). After standing for 5 days, these modes displayed excellent mechanical strength under various conditions, including under water and at low temperatures (**Figure** **7**a–c and Figures S33–S35, Supporting Information). Moreover, the 3D‐printed model is as time‐dependent as the cast poly(**TA**) as shown in Figure 7d,e. Time‐dependent tensile strength, Shore hardness, and modulus of the 3D‐printed mode strongly confirmed the typical self‐reinforcing phenomena of poly(**TA**). For example, the reduced moduli of the printed mode at 0.5 and 24 h are 0.69 and 1.26 GPa, respectively, as obtained from the time‐dependent nanoindentation tests. Importantly, the mechanical strength of 3D‐printing modes achieved an equilibrium state at approximate 7 days.

Poly(**TA**) materials doped with additives are 3D printable. Stimulus responsiveness was successfully maintained in the printed modes (Figures S36–S38, Supporting Information). For example, poly(**TA**), indicated by an arrow, containing 1,4‐bis‐(*α*‐cyano‐4‐methoxystyryl)‐2,5‐dimethoxybenzene, displayed typical and obvious fluorescent emission behavior under UV irradiation at 365 nm (Figure S36 and Video S10, Supporting Information). Moreover, poly(**TA**) with long afterglow materials were printed as underwater labels (Figure S38, Supporting Information).

To expand the application of poly(**TA**) materials, a 3D‐printed conductive children's insole (10 cm in length, 1.65 mm in thickness, right leg, doped with 5 wt% [lithium bis(trifluoromethanesulfonyl)imide] with an electrical output was selected as the pressure detector (Figure S39, Supporting Information). As shown in Figure 7g,h, external compression or tension is clearly monitored by this 3D‐printed insole, with a minimum detectable compression limit of 40 Pa. In the repeated weight loading/unloading test, no fatigue in the detection and output was observed (Figure S40, Supporting Information). This mode is also applicable at different temperature (10–35 °C) and humidity (10–99 RH%) (Figures S41–S42, Supporting Information).

### 2.5 Discussions

The starting point of this study lies in that: the realization of bottom‐up assembly in microscopic and macroscopic scales simultaneously. Thermally induced polymerization of **TA** to poly(**TA**) and 3D printing of poly(**TA**) were simultaneously achieved in the 3D‐printing process. **TA** displays three important features:

(a) The thermo‐/time‐dependent self‐assembly behavior of poly(**TA**) (bottom‐up assembly in microscopic scale).
(b) The effect of the time‐dependent assembly in the mechanical property of poly(**TA**).
(c) Poly(**TA**)/**TA** as the starting material in 3D FDM (bottom‐up assembly in macroscopic scale), due to the self‐reinforcing feature of poly(**TA**).

The time‐dependent assembly behavior of poly(**TA**) (Feature a) leads to the occurrence of self‐enhancement feature (mechanical strength increased within time) of poly(**TA**) (Feature b). Due to this unique property, poly(**TA**) was applied as the 3D FDM material (Feature c). In other words, “feature b” is the fruit of “feature a,” and “feature b” is the basic of “feature c.” If poly(**TA**) did not show the time‐dependent self‐reinforcing property, the newly printed modes would gradually collapse.

## 3 Conclusion

Inspired by the bottom‐up assembly of natural biological systems, an artificial additive manufacturing process was developed. Thermally induced self‐assembly patterns and 3D FDM‐printing technology were combined to construct poly(**TA**) molecular assemblies and fabricate bulk materials. The time‐dependent assembly of poly(**TA**) not only occurred on the microscopic scale but also exerted a great influence on the bulk assembly of 3D‐printed materials. 3D‐printable poly(**TA**) evolved from fragile and soft species via the spontaneous assembly behavior of poly(**TA**) and **TA**. Poly(**TA**) is a unique self‐reinforcing polymer material that shows great potential for 3D FDM printing and is an alternative 3D‐printable filament material.

## Conflict of Interest

The authors declare no conflict of interest.

## Supporting information

Supporting Information

Supporting Information Video 1

Supporting Information Video 2

Supporting Information Video 3

Supporting Information Video 4

Supporting Information Video 5

Supporting Information Video 6

Supporting Information Video 7

Supporting Information Video 8

Supporting Information Video 9

Supporting Information Video 10

## Data Availability

The data that support the findings of this study are available from the corresponding author upon reasonable request.
