# Supplementary material for: Poly(thioctic acid): From Bottom‐Up Self‐Assembly to 3D‐Fused Deposition Modeling Printing
Source: Adv Sci (Weinh). 2022 Oct 11;9(33):2203630. doi: 10.1002/advs.202203630 (PMC9685451; doi:10.1002/advs.202203630)
Supplement: Supplementary file 1 — Supporting Information [file ADVS-9-2203630-s006.pdf]

ADVANCED SCIENCE
Open Access

Supporting Information

for *Adv. Sci.*, DOI 10.1002/advs.202203630

Poly(thioctic acid): From Bottom-Up Self-Assembly to 3D-Fused Deposition Modeling Printing

Changyong Cai, Shuanggen Wu, Yunfei Zhang, Fenfang Li, Zhijian Tan and Shengyi Dong**

Supplementary Information

Poly(Thioctic Acid): from Bottom-Up Self-Assembly to 3D Fused Deposition Modeling Printing

Changyong Cai[1], Shuanggen Wu[1], Yunfei Zhang[1], Fenfang Li[2], Zhijian Tan[3*]& Shengyi Dong[1*]

Dr. C. Y. Cai, Dr. G. S. Wu, Dr. Y.F. Zhang, Prof. S. Y. Dong

College of Chemistry and Chemical Engineering, Hunan University, Changsha 410082, China

Prof. F.F. Li
College of Chemistry and Chemical Engineering, Central South University, Changsha 410083, China.

Prof. Z.J. Tan
Institute of Bast Fiber Crops, Chinese Academy of Agricultural Sciences, Changsha 410205, China;

*To whom correspondence should be addressed.
E-mail: dongsy@hnu.edu.cn; tanzhijian@caas.cn

Table of Contents

1. Materials and methods S3
2. Physical properties of poly(**TA**) S5
3. Time-dependent MALDI-TOF-MS spectra of poly(**TA**) S5
4. Time-dependent content of **TA** in poly(**TA**) S6
5. Time-dependent FT-IR spectra of **TA** and poly(**TA**) S7
6. Time-dependent powder X-ray diffraction (PXRD) patterns of **TA** and poly(**TA**) S7
7. Temperature-dependent powder X-ray diffraction (PXRD) pattern of **TA** and poly(**TA**) S8
8. Thermogravimetric analysis(TGA) of **TA** and poly(**TA**) S8
9. Differential scanning calorimeter (DSC) measurements of **TA** and poly(**TA**) S9
10. Model reactions in poly(**TA**) by simulation calculation S9
11. Time-dependent scanning electron microscopy (SEM) of poly(**TA**) S10
12. ^{1}H NMR spectra of **TA** and poly(**TA**) S12
13. Small angle X-ray scattering (SAXS) of **TA** and poly(**TA**) S12
14. Mechanical properties of poly(**TA**) S13
15. Time-dependent rheology measurements of poly(**TA**) S14
16. Time-dependent DMA tests of poly(**TA**) S15
17. Molecular dynamics simulation calculation S15
18. Tensile strength of poly(**TA**) with a variety of additives S16
19. Preparation of filaments S17
20. Viscosity of poly(**TA**) S17
21. 3D printing and application S18
22. Videos S25
23. Reference S26

1. Materials and methods

DL-thioctic acid (**TA**) was purchased from Shanghai Aladdin Biochemical Technology Co., Ltd (Shanghai, China). Other solvents and materials were commercially obtained and used directly. Nuclear magnetic resonance (NMR) spectra were acquired on a Bruker-AV400 MHz spectrometer. Infrared (IR) spectra were collected on a Thermo Scientific Nicolet iS10 FT-IR spectrometer. Powder X-ray diffraction (PXRD) spectra were collected on a Bruker D8 ADVANCE. Thermogravimetric analysis (TGA) was carried out using a TG 5500, and the heating rate was 10 ℃ min^{-1} from 30 to 600 ℃ in nitrogen atmosphere. Differential scanning calorimeter (DSC) measurements were obtained by a TAQ200 with a heating rate of 10 ℃in from -10 to 120 ℃ in nitrogen atmosphere. Scanning electron microscopy (SEM) images were collected on HITACHI UHR FE-SEM SU8010. Rheology measurements were performed on an Anton Paar MCR 92. The laminator model PP15 was chosen with 15 mm of diameter and 1 mm of gap. Mechanical strength was tested by universal testing machine (HT-101SC-5). Dynamic thermomechanical analyses (DMA) were performed on a DMA 8000-PerkinElmer using shear model. Matrixassisted laser desorption/ionization-time of flight mass spectrometry (MALDI-TOF-MS) was performed on a Bruker UltrafleXtreme TOF mass spectrometer in reflection mode with 2,5- dihydroxybenzoic acid (DHB) as matrix. Small angle X-ray scattering (SAXS) was performed on an Anton Paar SAXSess. Brunner−Emmet−Teller (BET) surface area analyses were obtained by Belsorp-max ASAP2460. Shore hardness was measured by a Shaos durometer (type D, ChuanLu). Electron spray ionization (ESI) mass analysis was performed on Thermo Scientific Q Exactive. Electric response measurements were conducted on electric conductor parameter meter (Keithley 2450). Nanoindentation were obtained by a Hysitron TI 950.

Preparation of poly(TA)

TA powder (and additives) was heated at 120 ℃.[1]

Preparation of poly(TA) filaments

TA powder was added to the injection molding machine (Wellzoom, China) and processed into filaments at 100 ℃. Filaments were cooled by ice water and collected

with self-made collection equipment.

3D Fused Deposition Modeling (FDM) printing

The filament printing method and viscous melts method were used in the 3D printing. Filament printing method was applied by a short range extrusion 3D printer (MagicMaker, China) or a 3D printing pen. The printing temperature of the extruder is 100-120 ℃, and the printing speed is 10 mm s^{-1}. The printing temperature of the 3D printing pen is 130 ℃. The printing temperature of viscous melts method was 100 ℃ (LOBO, China). The models were created by CAD software (AutoCAD, Autodesk) and sliced by ChiTu slicer software.

Synthesis of 1,4-Bis-(α-cyano-4-methoxystyryl)-2,5-dimethoxybenzene

The synthesis route is based on a reported method.[2]

Determination of TA content

Contents of **TA** were measured by a Dalian Elite series high performance liquid chromatography (HPLC) (iChrom 5100, China). A Kromasil C_{18} chromatographic column (250 × 4.6 mm, particle size 5 μm) was used for the sample analysis. The mobile phase was methanol and aqueous solution with 0.1 % acetic acid (v/v=54/46). The flow rate was 1.0 mL min^{-1}, the detection wavelength was 322 nm, and the column temperature was 30 ℃. The standard curve for analysis of **TA** is shown in Eq. (1).

$$Y = 762.05\,X + 1.698 \quad R^2 = 0.9991 \qquad (1)$$

Where Y is the peak area of **TA** and X is the concentration of **TA**. The standard solution concentration range of **TA** was 0.02−0.05 mg mL^{-1}.

Simulation calculation

Gibbs free energy calculations were carried out with the Gaussian 16 software.[3] The M06-2X functional was adopted for all calculations.[4] For geometry optimization and frequency calculations, the def2-SVP basis set was used, and the optimal geometry for each compound was determined.[4, 5] The singlet point energy calculations were performed with a larger basis set def2-TZVP basis set.[4, 5] The DFT-D3 dispersion correction was applied to correct the weak interaction to improve the calculation accuracy.[6]

All-atom molecular dynamics simulations were used in the current work. Five cases

were designed in Table S3. Stretching, shock, and interaction were simulated. Structural model of poly(**TA**) with **TA** and a polymerization degree of 20 and 30 were established on the basis of the Materials Studio (MS) and Large-scale Atomic/Molecular Massively Parallel Simulator (LAMMPS) software package.[7] The time step was 1 fs and the cutoff distance is 9.5 Å for our simulations. Compass and Dreiding force field were applied to poly(**TA**) to describe the interaction.[8]

Statistical Analysis

All the results in this study were presented as mean ± S.D.. The statistical analyses were carried out with the GraphPad prism 8.3 software.

2. Physical properties of poly(TA)

Table S1. Physical properties of poly(**TA**) with time-dependent.

Density (120 h)	1.2947 g cm^{-3}
Colour	yellow
Thermal stability	>180 ℃
BET (30 min)	0.4541 m^2 g^{-1}
BET (120 h)	0.4213 m^2 g^{-1}

3. Time-dependent MALDI-TOF-MS spectra of poly(TA)

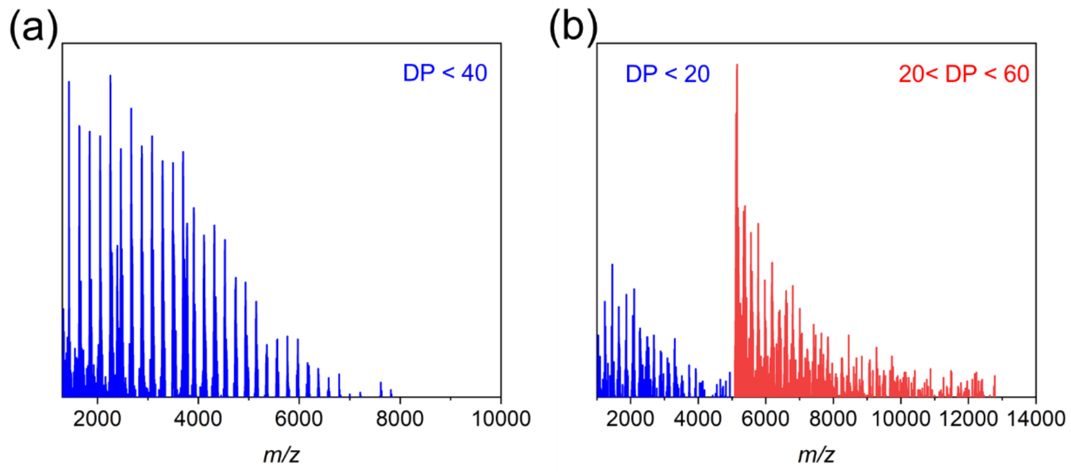

Figure S1. Time-dependent MALDI-TOF-MS spectra of poly(**TA**): (a) 24 h; (b) 168 h.

4. Time-dependent content of TA in poly(TA)

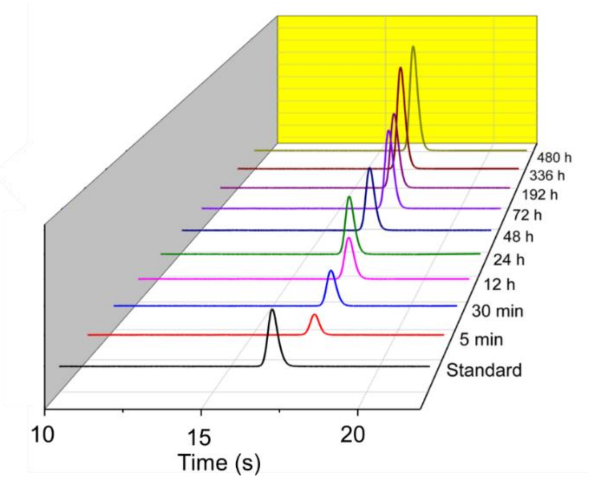

Figure S2. HPLC spectra of **TA** in poly(**TA**).

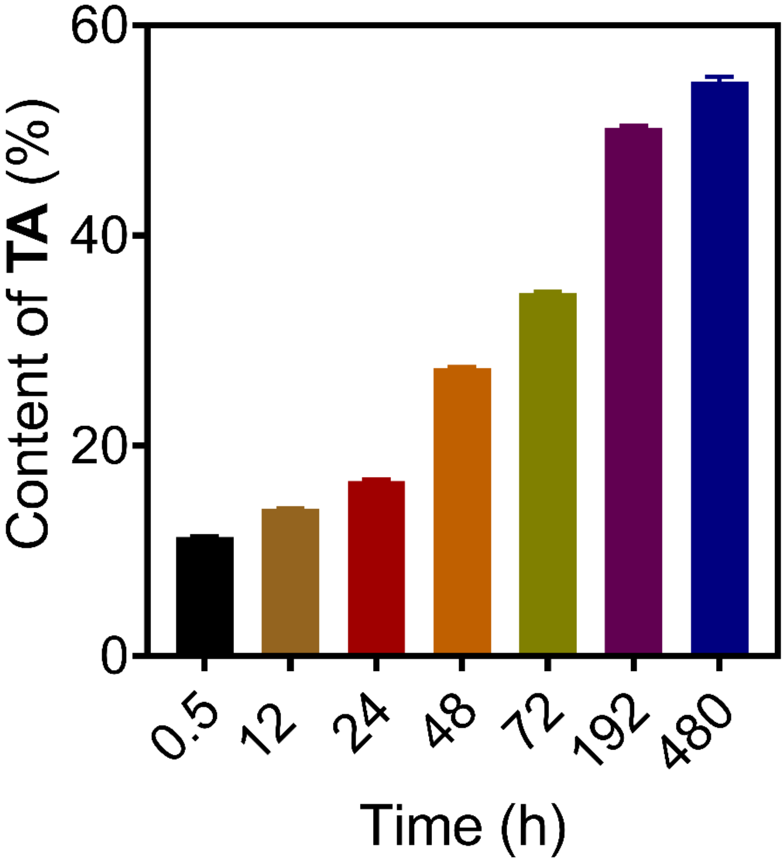

Figure S3. Time-dependent contents of **TA** in poly(**TA**) (means ± S.D., n = 3).

5. Time-dependent Fourier-Transform IR (FT-IR) spectra of TA and poly(TA)

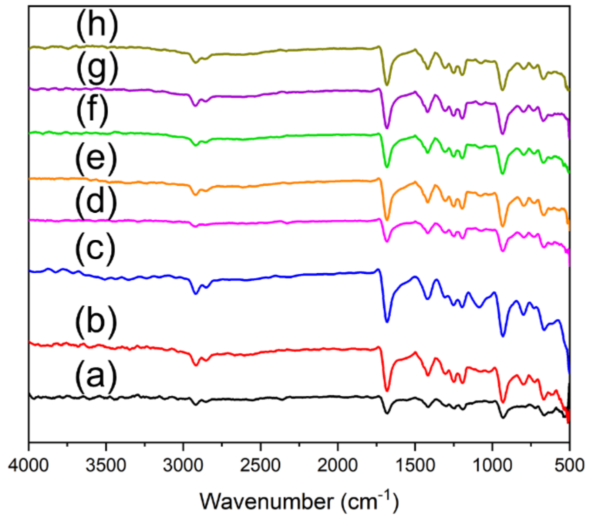

Figure S4. Time-dependent FT-IR spectra of **TA** and poly(**TA**): (a) **TA**; (b) 2 h; (c) 24 h; (d) 48 h; (e) 96 h; (f) 168 h; (g) 216 h; (h) 312 h.

6. Time-dependent powder X-ray diffraction (PXRD) patterns of TA and poly(TA)

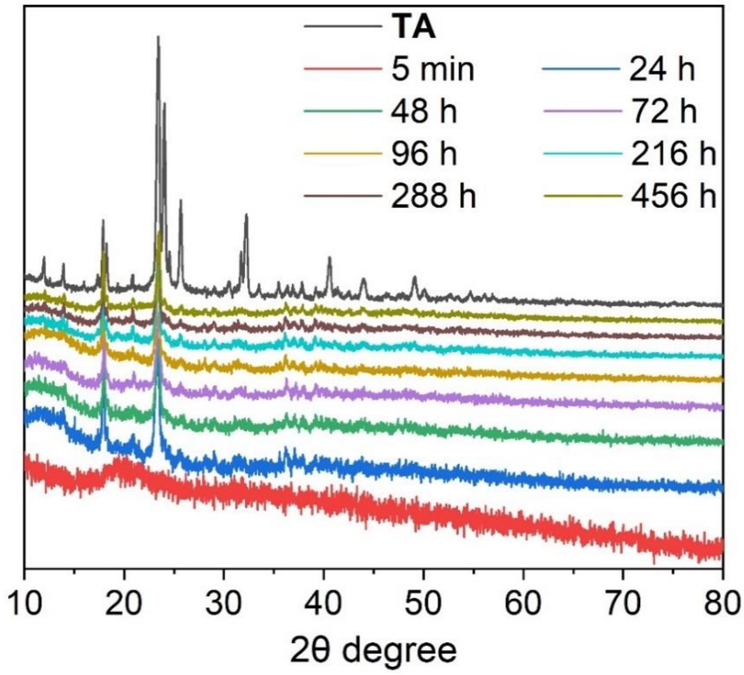

Figure S5. Time-dependent PXRD spectra of **TA** and poly(**TA**).

7. **Temperature-dependent powder X-ray diffraction (PXRD) patterns of TA and poly(TA)**

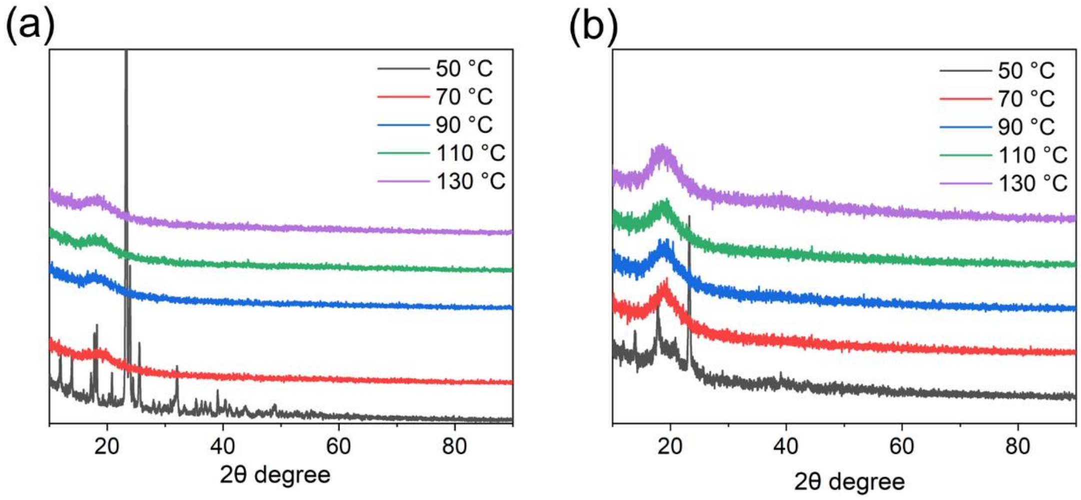

Figure S6. Temperature-dependent PXRD pattern of (a) **TA** and (b) poly(**TA**) (144 h).

8. **Thermogravimetric analysis (TGA) of TA and poly(TA)**

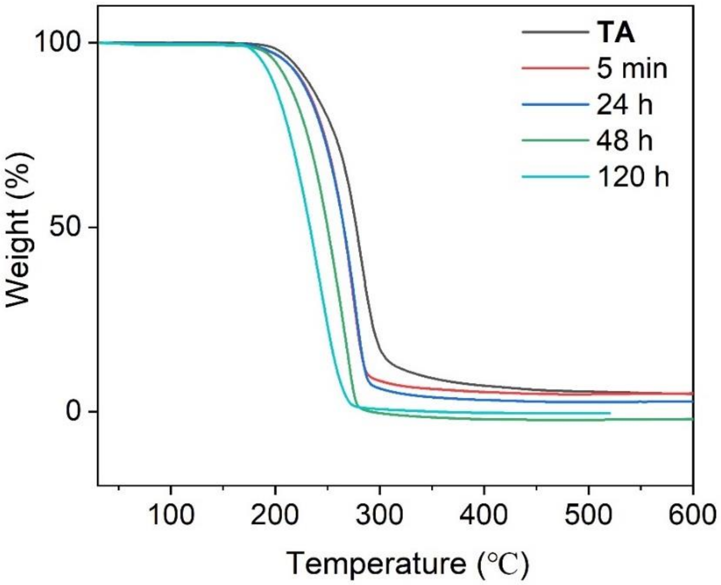

Figure S7. TGA spectra of **TA** and poly(**TA)** with time-dependent (5 min–120 h).

9. Differential scanning calorimeter (DSC) measurements of poly(TA)

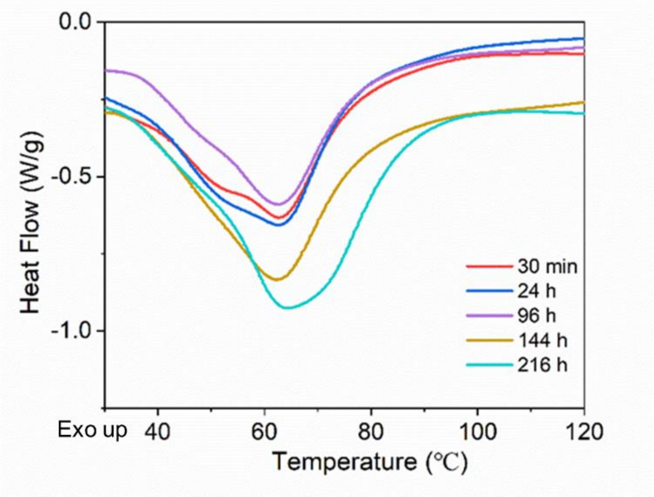

Figure S8. DSC spectra of poly(**TA**).

10. Model reactions in poly(TA) by simulation calculation

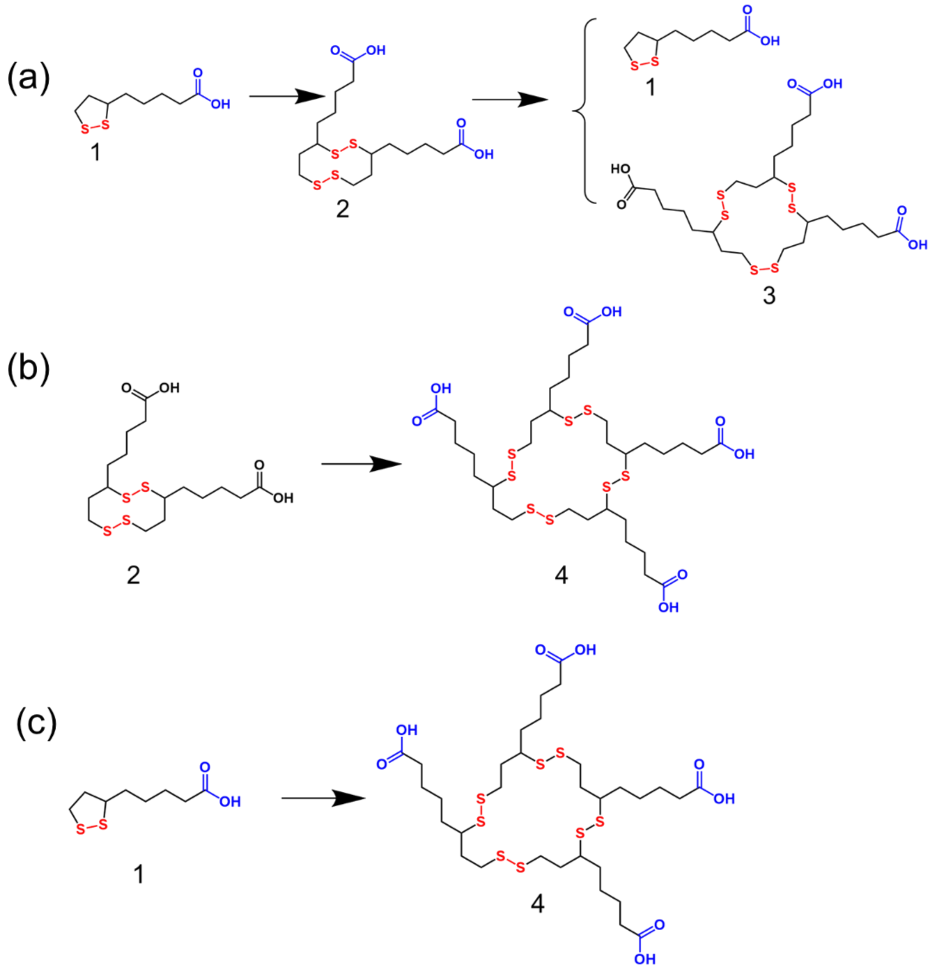

Figure S9. Design of model reactions: (a) dimer and trimmer formations; (b) tetramer formation from dimer; (c) tetramer formation from **TA**.

Table S2. Gibbs free energy of the reaction processes.

	**Δ*G*（kJ mol^{-1}）**
1+1=2	3.81
2+2=3+1	-46.77
2+2=4	-71.40
1+1+1+1=4	-63.77

11. Time-dependent scanning electron microscopy (SEM) of poly(TA)

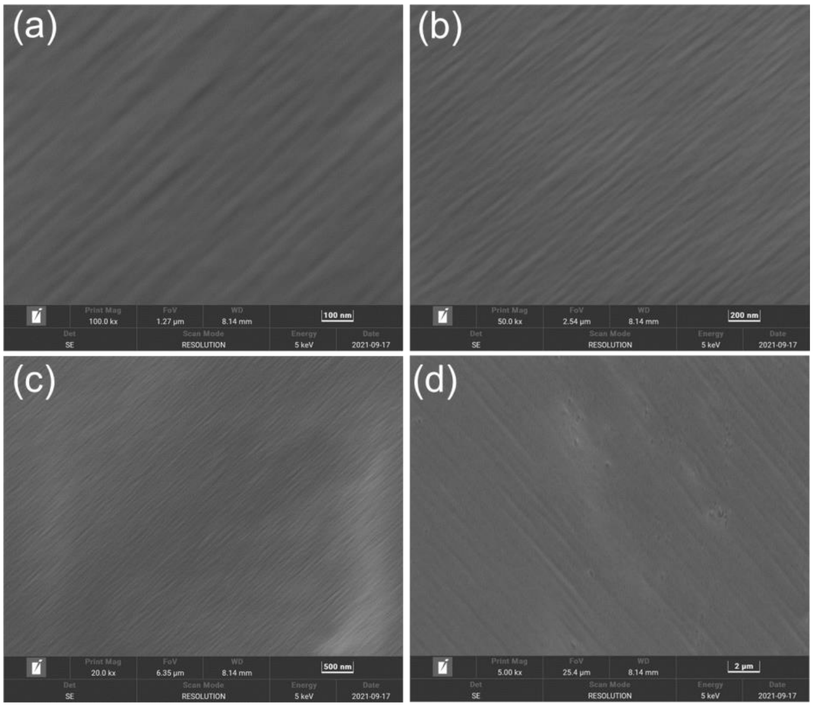

Figure S10. SEM images of poly(**TA**) (a–d) (30 min).

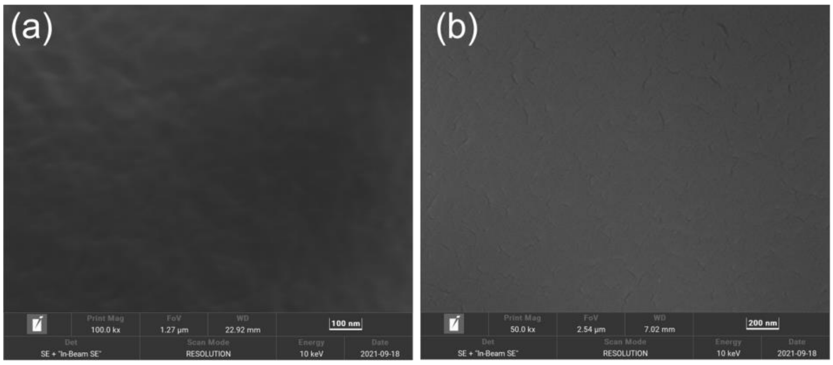

Figure S11. SEM images of poly(**TA**) (a–b) (24 h).

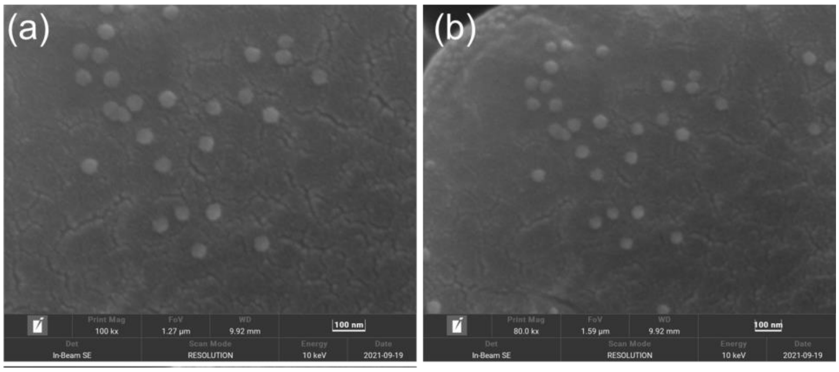

Figure S12. SEM images of poly(**TA**) (a–b) (48 h).

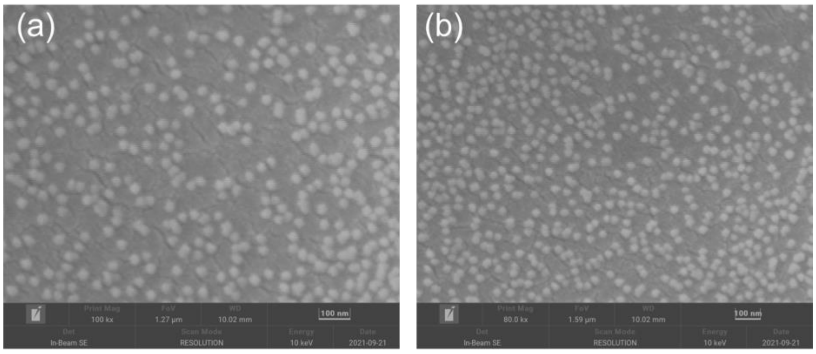

Figure S13. SEM images of poly(**TA**) (a–d) (96 h).

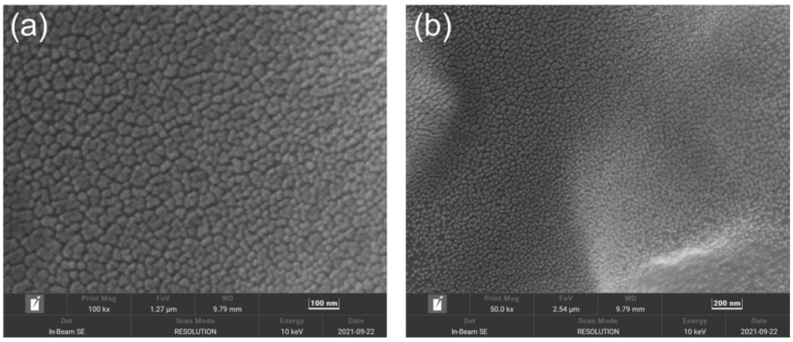

Figure S14. SEM images of poly(TA) (a–d) (120 h).

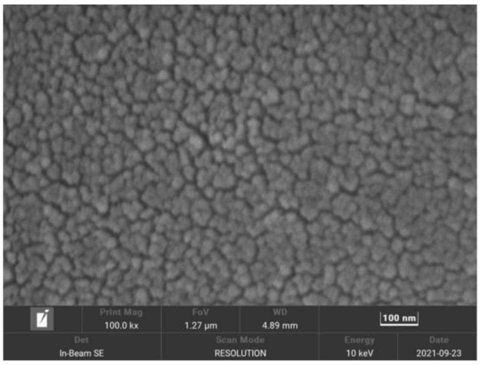

Figure S15. SEM image of poly(**TA**) (a–d) (144 h).

12. ^{1}H NMR spectra of TA and poly(TA)

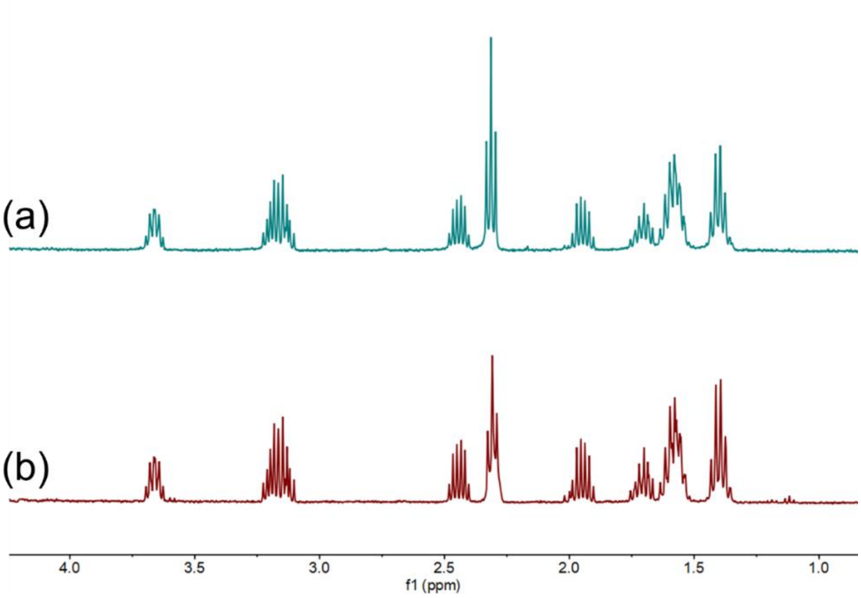

Figure S16. ^{1}H NMR spectra of **TA** and poly(**TA**) (48 h) (D_2O, room temperature): (a) **TA**; (b) poly(**TA**).

13. Small angle x-ray scattering (SAXS) of poly(TA)

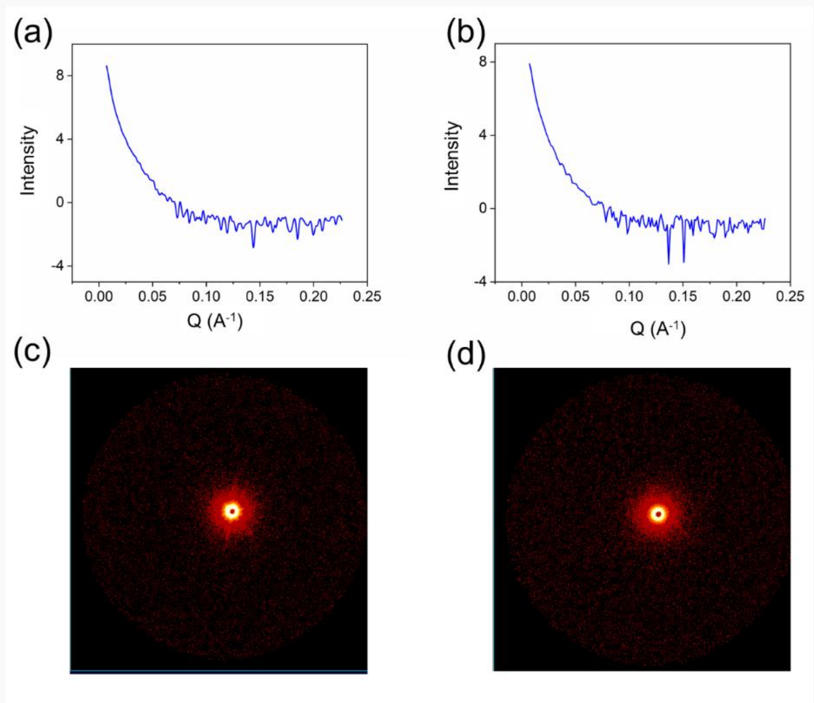

Figure S17. SAXS patterns of **TA** and poly(**TA**): (a) 1D SAXS of **TA**; (b) 1D SAXS of poly(**TA**) (120 h); (c) 2D SAXS of **TA**; (d) 2D SAXS of poly(**TA**) (120 h).

14. Time-dependent mechanical properties of poly(TA)

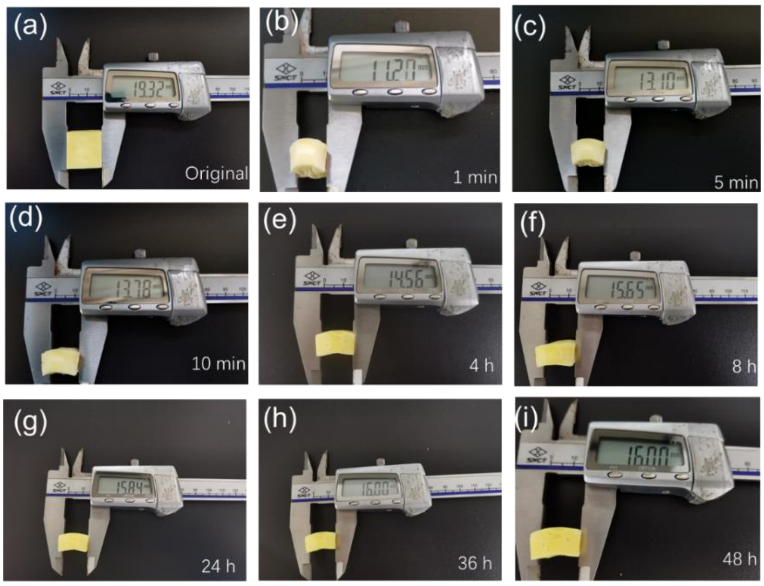

Figure S18. Photos of poly(**TA**) after compression with 10000 N: (a) original poly(**TA**) (120 h); (b) 1 min after compression; (c) 5 min after compression; (d) 10 min after compression; (e) 4 h after compression; (f) 8 h after compression; (g) 24 h after compression; (h) 36 h after compression; (i) 48 h after compression.

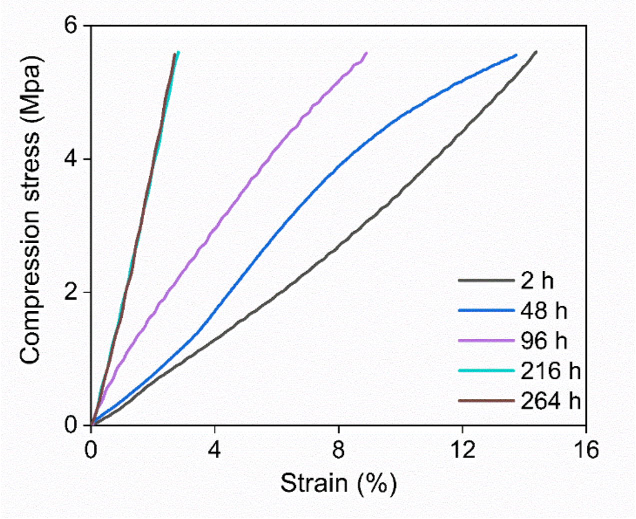

Figure S19. Temperature-dependent compression tests of poly(**TA**).

15. Time-dependent rheology measurements of poly(TA)

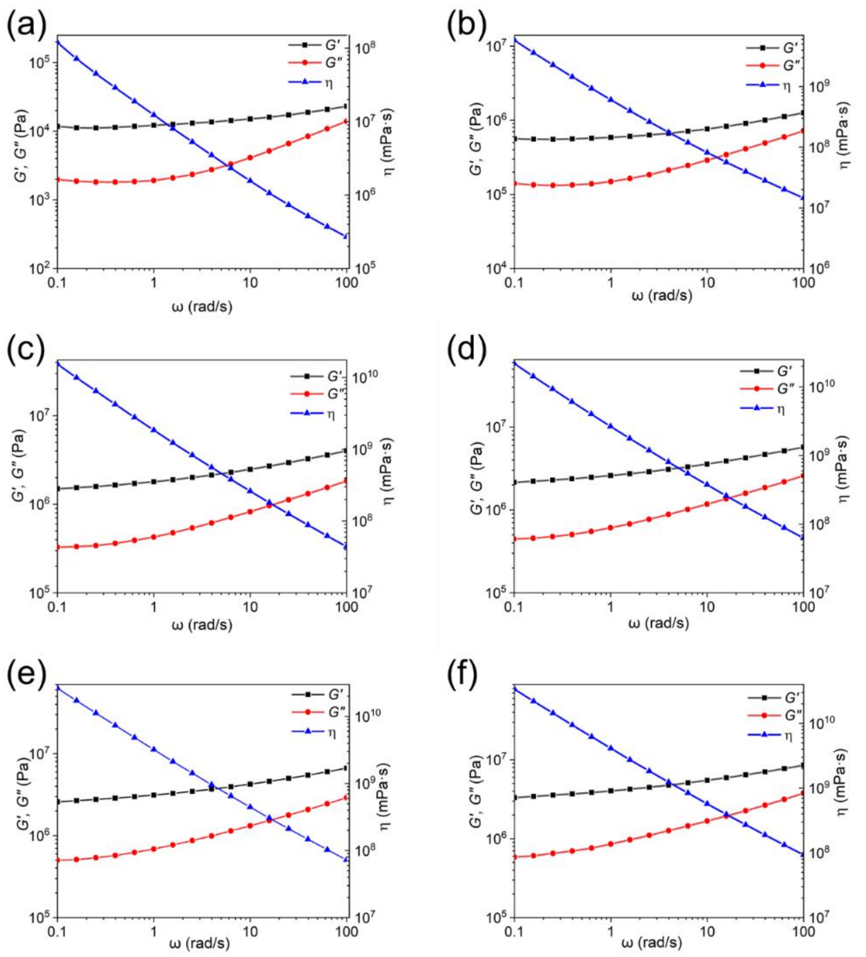

Figure S20. Time-dependent storage modulus (*G'*), loss modulus (*G''*) and complex viscosity (η) value of poly(**TA**): (a) 30 min; (b) 12 h; (c) 24 h; (d) 48 h; (e) 96 h (f) 216 h.

16. Time-dependent DMA tests of poly(TA)

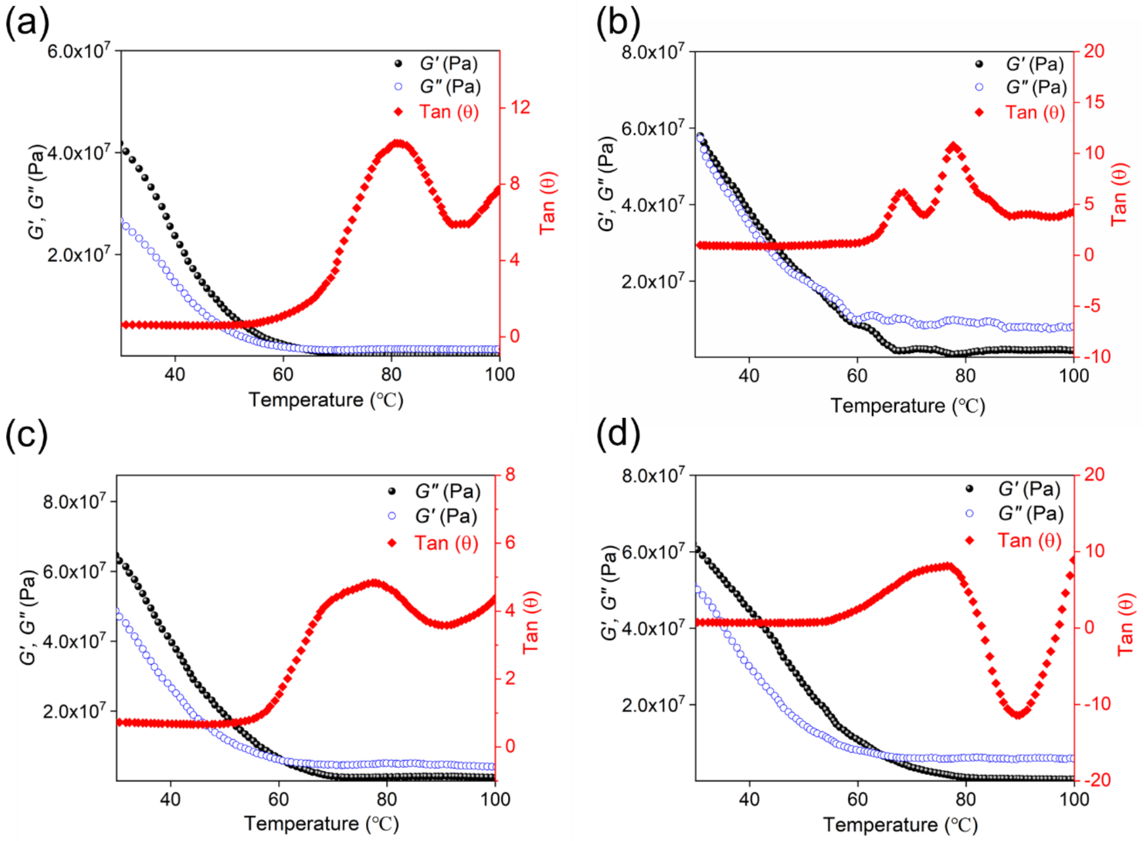

Figure S21. Time-dependent DMA tests of poly(**TA**): (a) 24 h; (b) 96 h; (c) 240 h; (d) 336 h.

17. Molecular dynamics simulation calculation

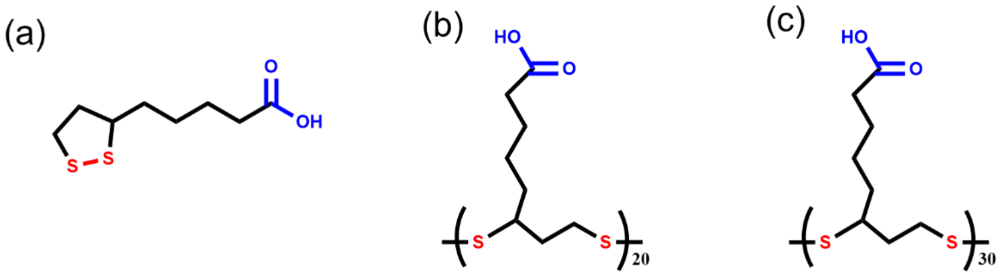

Figure S22. Design of model compounds for (a) **TA**, (b) 20 repeated **TA**, and (c) 30 repeated **TA**.

Table S3. Structural composition and energy distribution of three polymerization situation.

	Situation 1	Situation 2	Situation 3	**TA**
Structure a			1000	3000
Structure b	150		25	

Structure c		100	50	
Non-bond energy (kcal mol^{-1})	-65067.4	-58377.7	-70732	-19065.195
Hydrogen bond (kcal mol^{-1})	-3333.35	-3211.86	-3605.71	-1076.887
van van Waals (kcal mol^{-1})	2132.128	2169.544	-506.827	-1454.344
Electrostatic (kcal mol^{-1})	-63866.1	-57335.4	-66619.4	-16533.964

18. Tensile strength of poly(TA) with a variety of additives

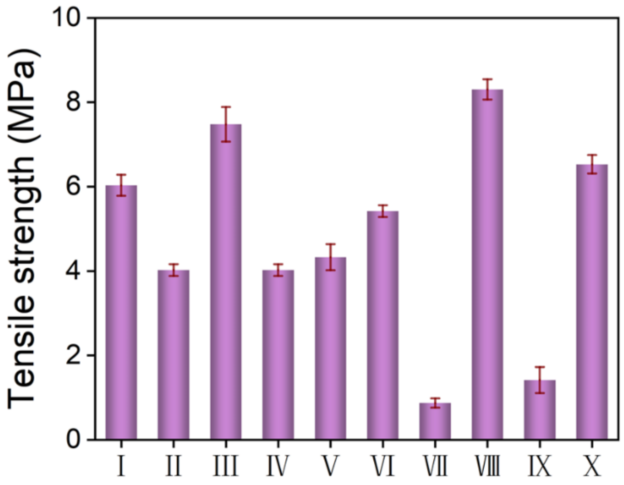

Figure S23. Tensile strength of poly(**TA**) with additives (7 days) [I: no additive; II: nickel chloride hexahydrate (5 wt%); III: azure B (5 wt%); IV: sudan II, BS (5 wt%); V: acid fuchsin sodium salt (5 wt%); VI: gallium (20 wt%); VII: cobalt chloride (5 wt%); VIII: magnetic Fe_3O_4 nanoparticles (20 wt%); IX: photochromic material (20 wt%); X: 1,4-bis-(α-cyano-4-methoxystyryl)-2,5-dimethoxybenzene (0.1 wt%)]. (± S.D., n = 3).

19. Preparation of filaments

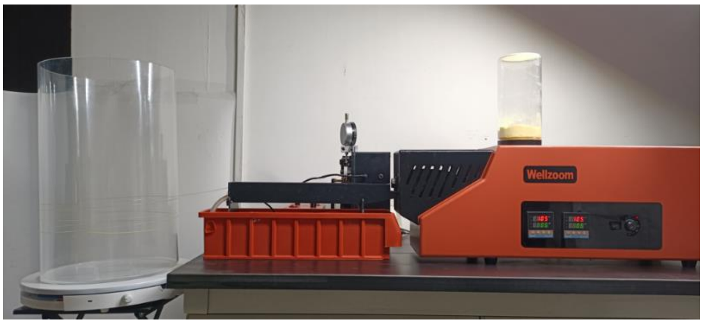

Figure S24. Photo of the filament extrusion process.

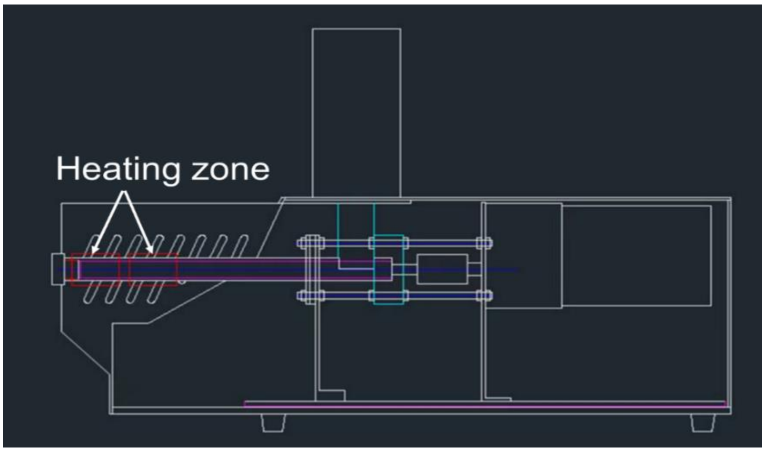

Figure S25. Cartoon of the filament extrusion process.

20. Viscosity of poly(TA)

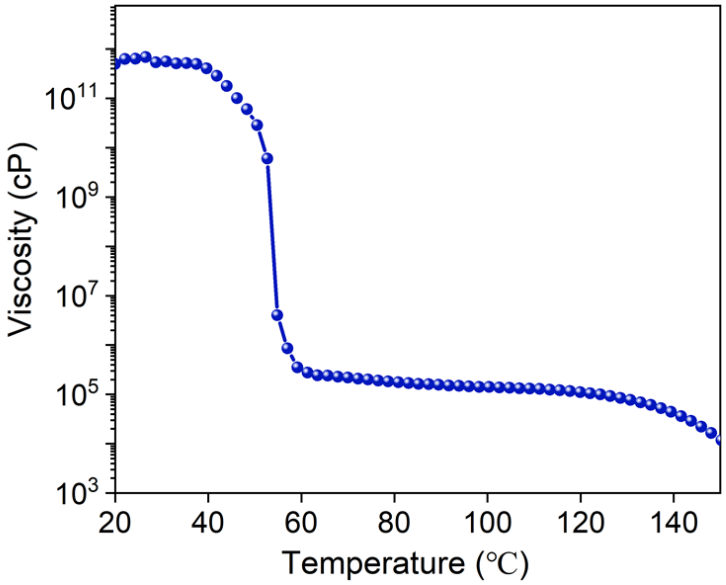

Figure S26. Temperature-dependent viscosity of poly(**TA**).

21. 3D printing and application

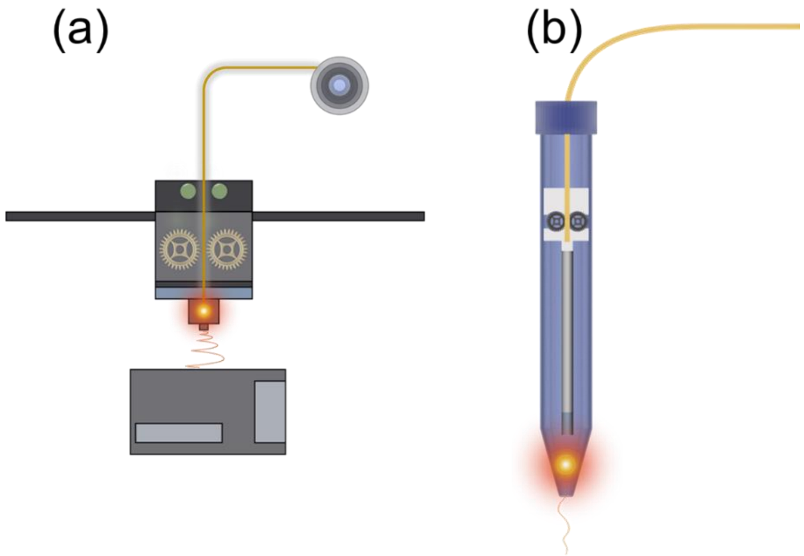

Figure S27. Cartoon of 3D printer printing process: (a) short range extrusion 3D printer; (b) 3D printing pen.

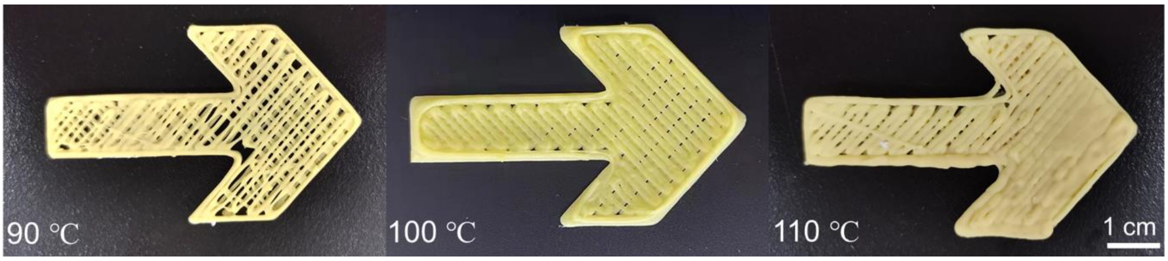

Figure S28. 3D printing of poly(**TA**) at different temperatures.

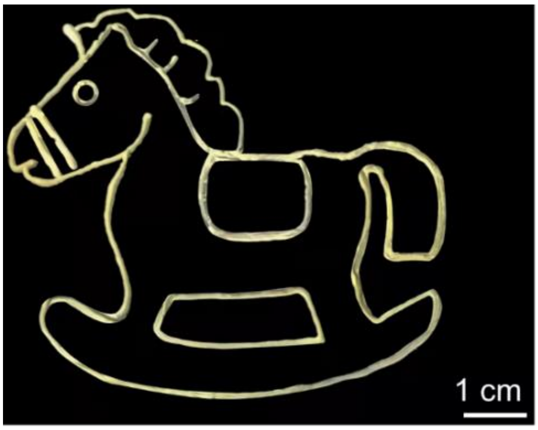

Figure S29. 3D printing pattern of poly(**TA**) by 3D printing pen.

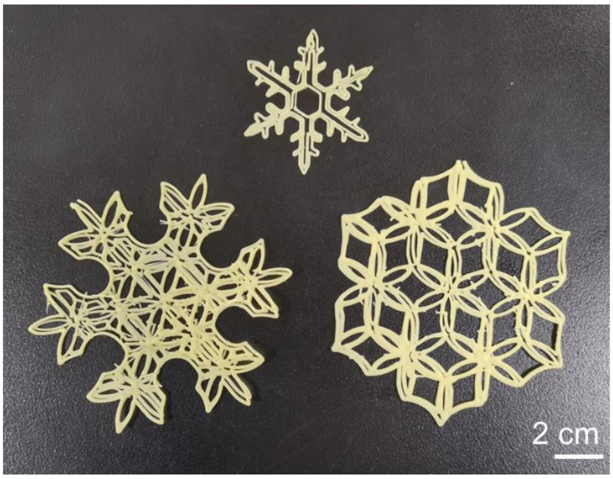

Figure S30. 3D printing of snowflakes.

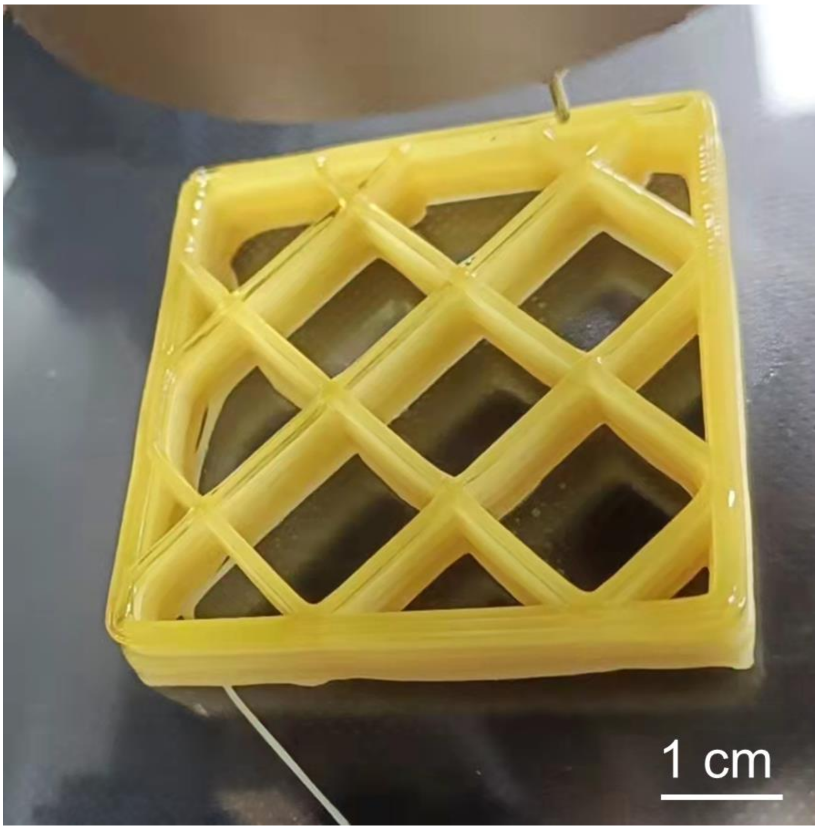

Figure S31. 3D printing of hollow cuboid.

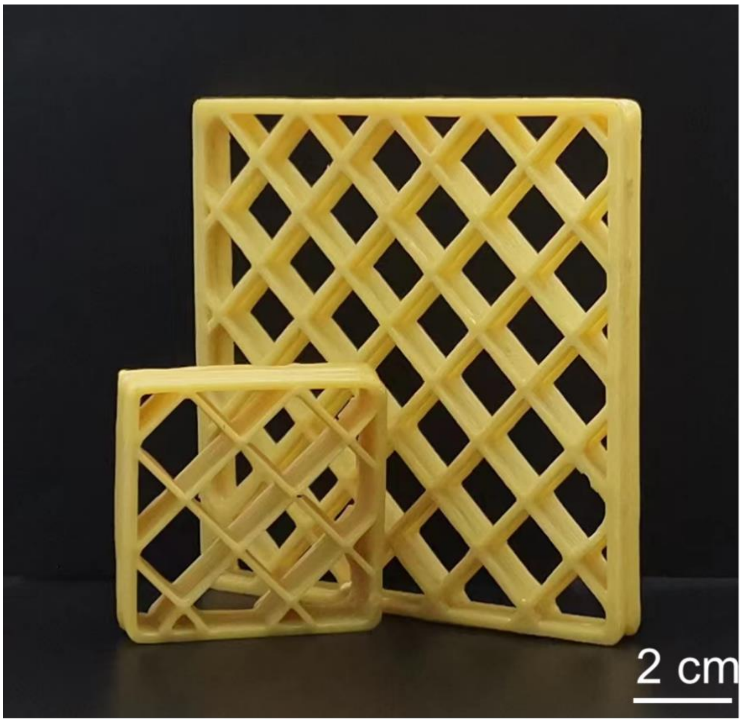
2 cm

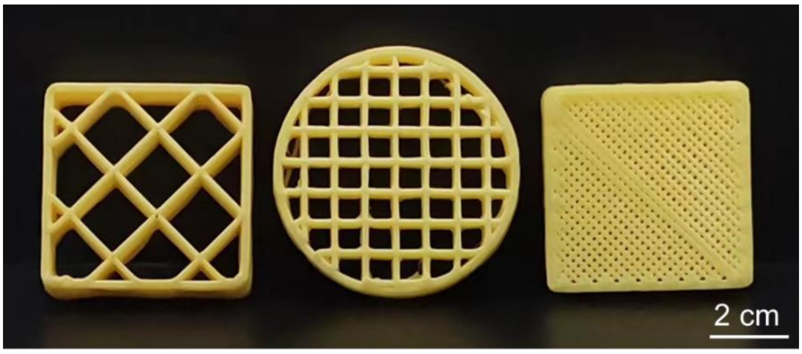
2 cm

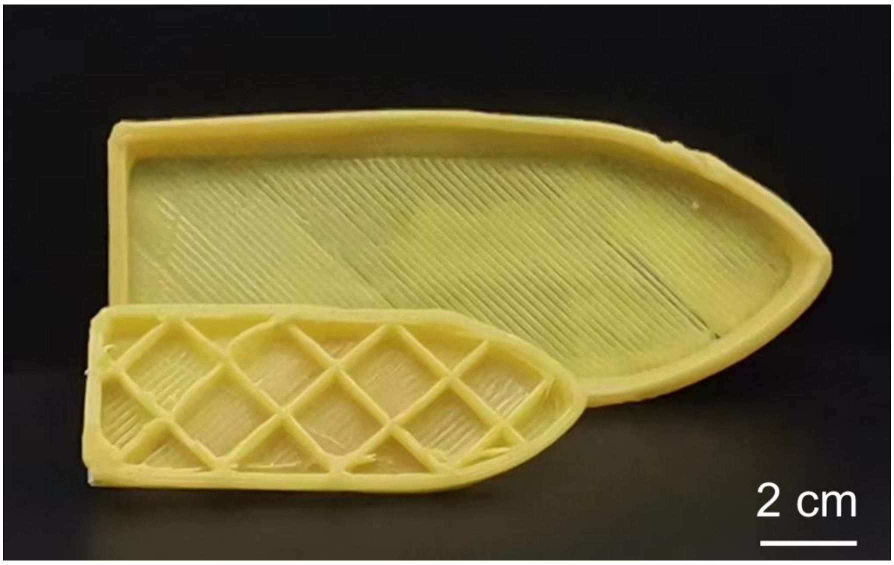
2 cm

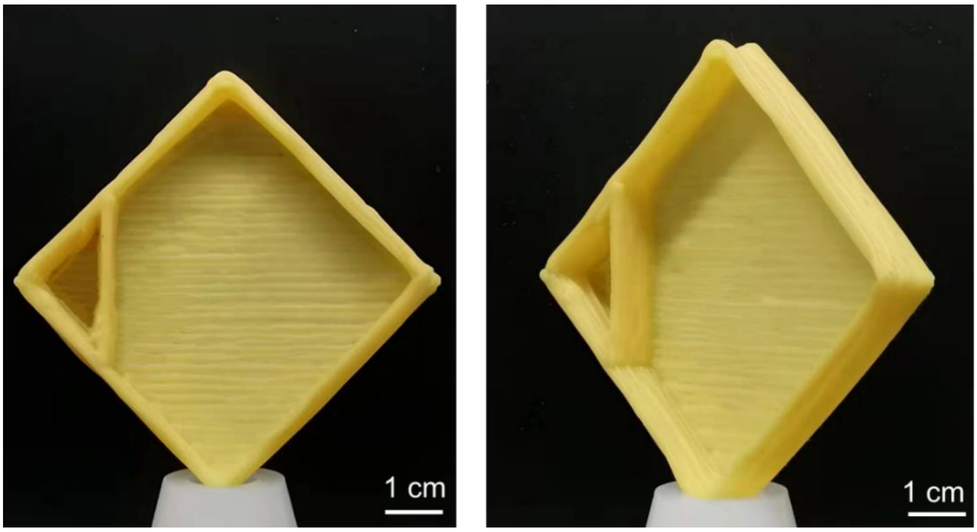

Figure S32. 3D printing of models.

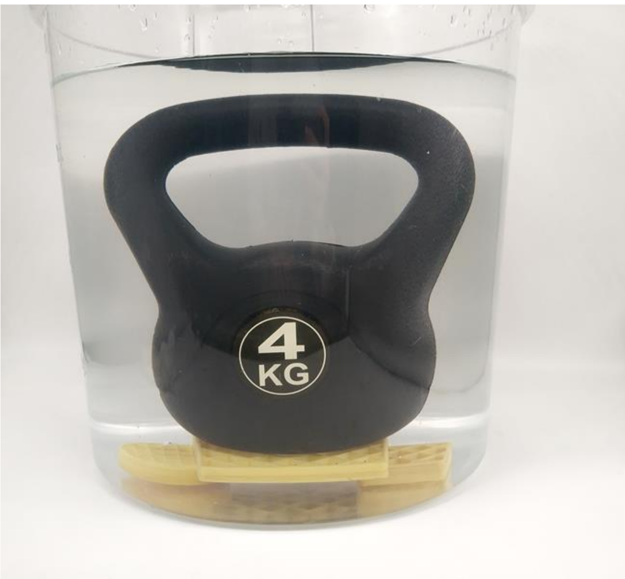

Figure S33. Under water weight loading test of 3D printed model.

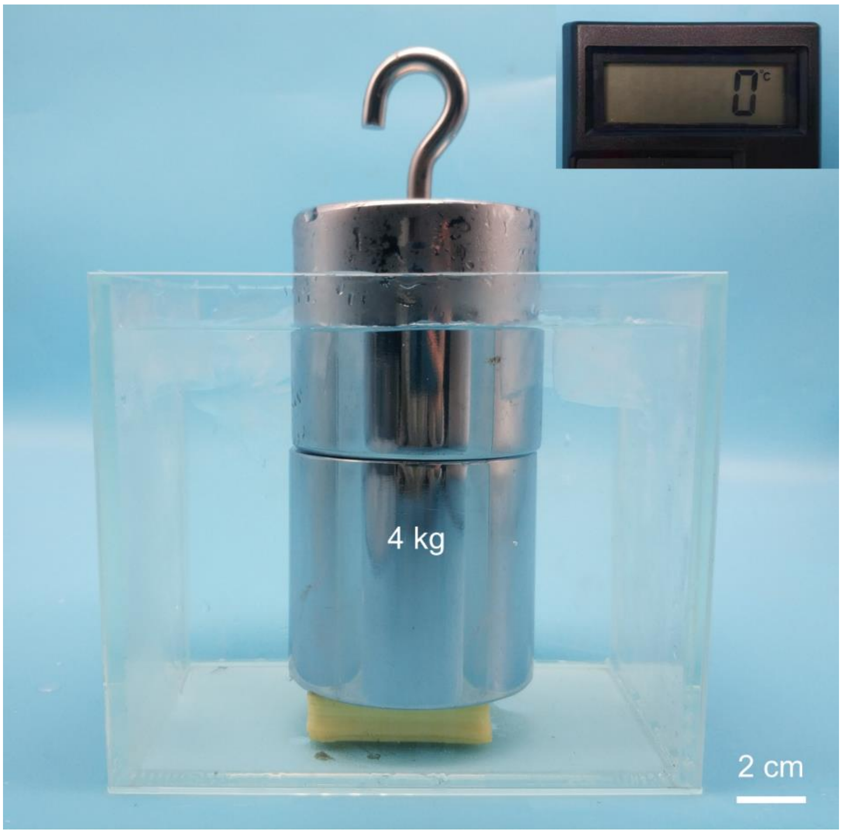

Figure S34. Under ice water weight loading test of 3D printed model.

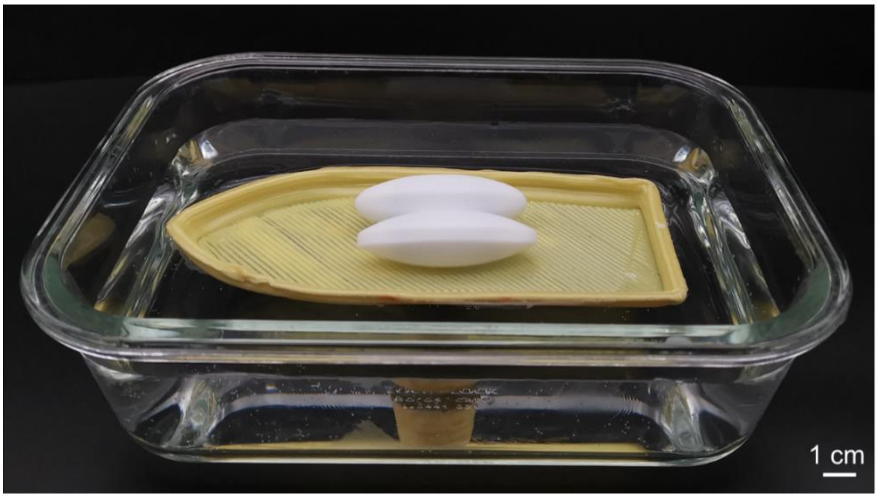

Figure S35. 3D printed boat on water loading 42.6 g.

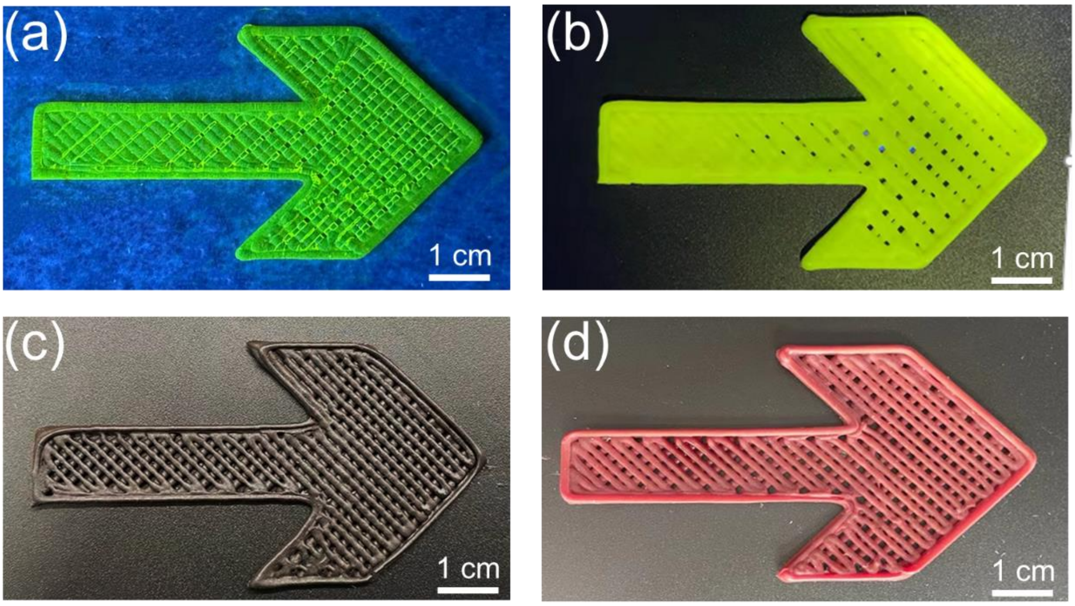

Figure S36. 3D print mode different additives: (a) with 1,4-bis-(α-cyano-4-methoxystyryl)-2,5-dimethoxybenzene (0.1 wt%)at 80 ℃; (b) with 1,4-bis-(α-cyano-4-methoxystyryl)-2,5-dimethoxybenzene (0.1 wt%) at room temperature; (c) with magnetic Fe_3O_4 nanoparticles (20 wt%); (d) with middle: sudan II, BS (5 wt%).

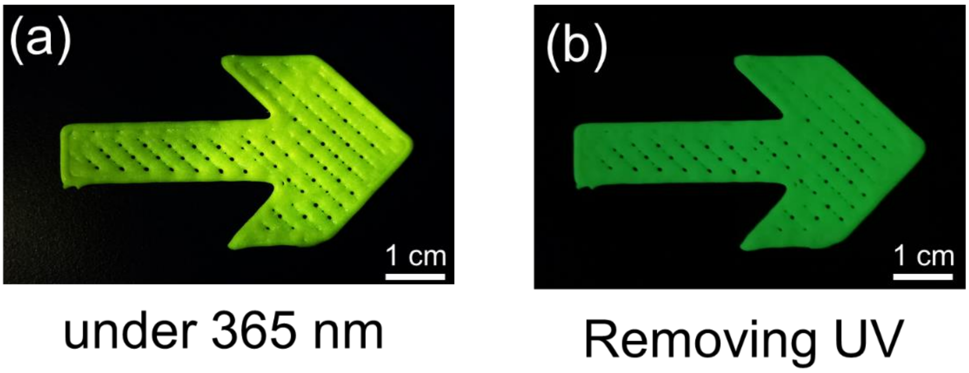

Figure S37. 3D printed mode with long afterglow $SrAl_2O_4$:Eu (10 wt%).

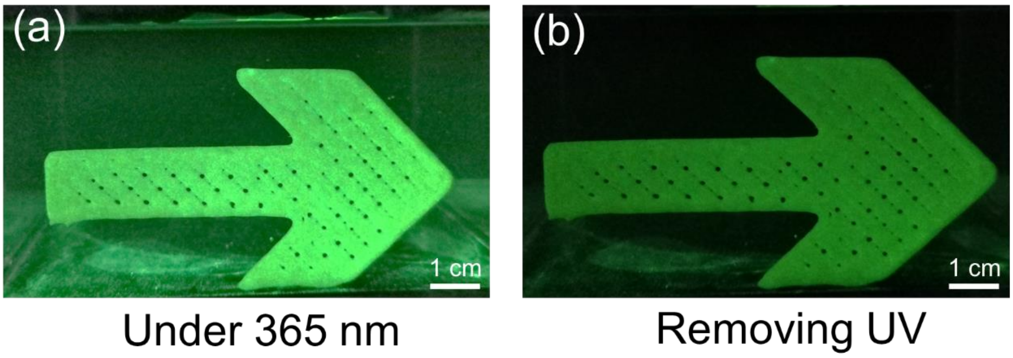

Figure S38. 3D printed mode with long afterglow material $SrAl_2O_4$:Eu (10 wt%) under water.

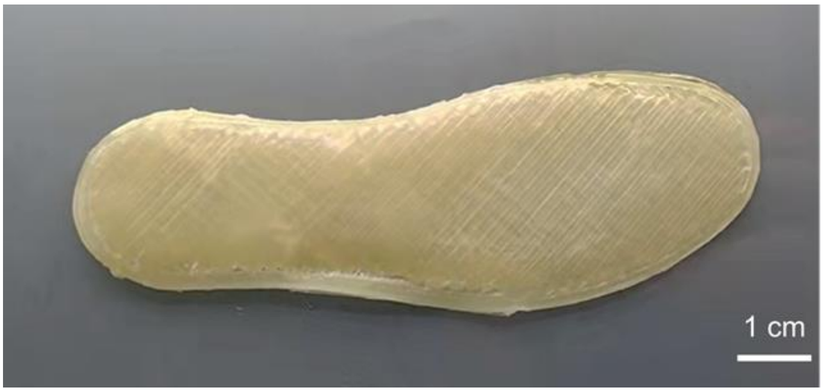

Figure S39. 3D printing insole with $LiNTf_2$ (5 wt%).

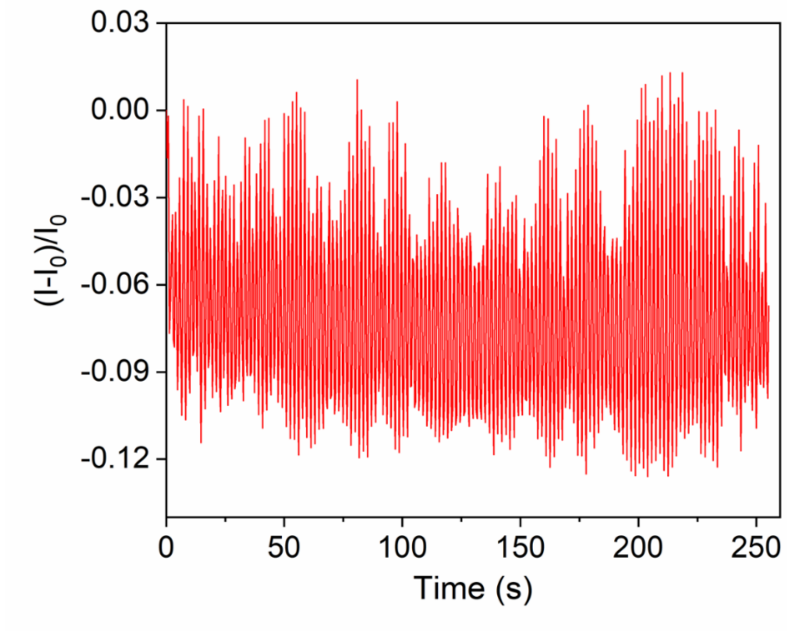

Figure S40. Stable relative current variation of the strain (50%) sensor over 300 cycles of loading/unloading.

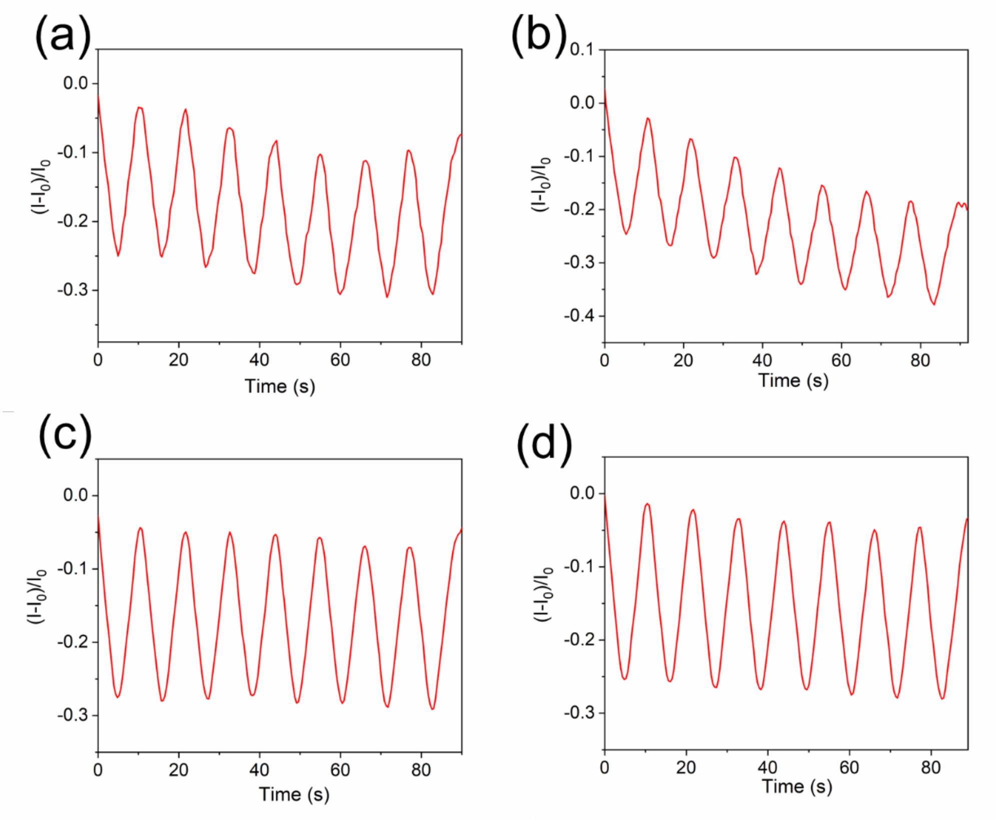

Figure S41. Stable relative current variation of the strain (50%) sensor over a wide humidity with multiple cycles of loading/unloading: (a) 10 RH%; (b) 30 RH%; (c) 70 RH%; (d) 90 RH%.

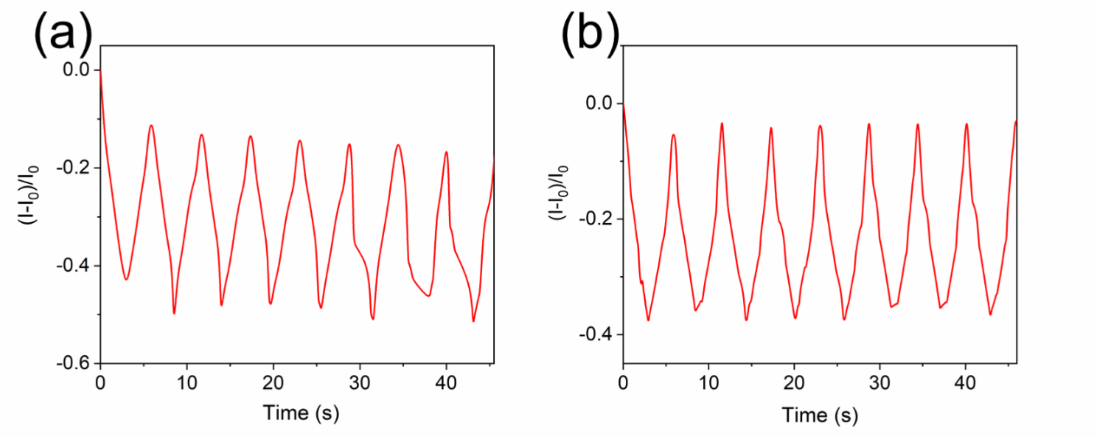

Figure S42. Stable relative current variation of the strain (50%) sensor over a wide temperature with multiple cycles of loading/unloading：(a) 10 °C; (b) 35 °C.

22. Videos

Video S1. Poly(**TA**) hemisphere pressed by a car.
Video S2. Poly(**TA**) ball was being thrown on the floor.
Video S3. The shock resistance of poly(**TA**).

Video S4. The anti-stretching capacity of poly(**TA**).
Video S5. Preparation of the filament.
Video S6. Repeated compression and tensile phenomena of poly(**TA**) spring.
Video S7. Poly(**TA**) fiber squeezed from the 3D printing pen.
Video S8. Model by viscous melts method.
Video S9. Model by filament printing method (short range extrusion 3D printer).
Video S10. Poly(**TA**) with 1,4-bis-(α-cyano-4-methoxystyryl)-2,5-dimethoxybenzene model under UV irradiation by viscous melts method.

23. Reference

[1] Q. Zhang, C.-Y. Shi, D.-H. Qu, Y.-T. Long, B. L. Feringa, H. Tian, *Sci. Adv.* **2018**, 4, eaat8192.

[2] C. Lwe, C. Weder, *Synthesis* **2002**, 9,1185.

[3] M. J. Frisch, G. W. Trucks, H. B. Schlegel, G. E. Scuseria, M. A. Robb, J. R. Cheeseman, G. Scalmani, V. Barone, B. Mennucci, G. A. Petersson, *Gaussian 09, Revision B.01.* **2010**.

[4] Z. Yan, D. G. Truhlar, *Theor. Chem. Acc.* **2008,** 119, 525.

[5] F. Weigend, R. Ahlrichs, *Phys. Chem. Chem. Phys.* **2005**, 7, 3297.

[6] F. Weigend, *Phys. Chem. Chem. Phys.* **2006**, 8, 1057.

[7] S. Grimme, J. Antony, S. Ehrlich, H. Krieg, *J. Chem. Phys.* **2010**, 132, 154104.

[8] a) J. Yang, X. Tang, F. Tian, T. Xu, C. Xie, G. Liu, S. Xie, L. Li, *J. Rheol.* **2019**, 63, 939; J.-F. Yin, Z. Zheng, J. Yang, Y. Liu, L. Cai, Q.-Y. Guo, M. Li, X. Li, T. L. Sun, G. X. Liu, C. Huang, S. Z. D. Cheng, T. P. Russell, P. Yin, *Angew. Chem. Int. Ed.* **2021**, 60 , 4894; c) J.-F. Yin, H. Xiao, P. Xu, J. Yang, Z. Fan, Y. Ke, X. Ouyang, G. X. Liu, T. L. Sun, L. Tang, S. Z. D. Cheng, P. Yin, *Angew. Chem. Int. Edt.* **2021,** 60, 22212; d) B Vu Tan,. A. Abdelrasoul, D. W. McMartin, *Comp. Mater. Sci.* **2021**, 187, 110079.
